# The Potential of High Voltage Discharges for Green Solvent Extraction of Bioactive Compounds and Aromas from Rosemary (*Rosmarinus officinalis* L.)—Computational Simulation and Experimental Methods

**DOI:** 10.3390/molecules25163711

**Published:** 2020-08-14

**Authors:** Marinela Nutrizio, Jasenka Gajdoš Kljusurić, Zvonimir Marijanović, Igor Dubrović, Marko Viskić, Elena Mikolaj, Farid Chemat, Anet Režek Jambrak

**Affiliations:** 1 Faculty of Food Technology and Biotechnology, University of Zagreb, 10000 Zagreb, Croatia; jasenka.gajdos@pbf.hr (J.G.K.); elena.mikolaj@yahoo.com (E.M.); 2 Faculty of Chemistry and Technology, University of Split, 21000 Split, Croatia; zvonimir.marijanovic@ktf-split.hr; 3 Teaching Institute of Public Health of the Primorsko-goranska County, 51000 Rijeka, Croatia; igor.dubrovic@zzjzpgz.hr; 4 Faculty of Agriculture, University of Zagreb, 10000 Zagreb, Croatia; mviskic@agr.hr; 5 Université d’Avignon et des Pays du Vaucluse, 84000 Avignon, France; farid.chemat@univ-avignon.fr

**Keywords:** high voltage electrical discharge, rosemary, COSMO–RS, Hansen solubility parameters, bioactive compounds, food aromas, extractions

## Abstract

Rosemary (*Rosmarinus officinalis* L.) is a Mediterranean medicinal and aromatic plant widely used due to valuable bioactive compounds (BACs) and aromas. The aim of the study was to evaluate the extraction of intracellular compounds from rosemary combining experimental procedure by means of high voltage electrical discharge (HVED), with a theoretical approach using two computational simulation methods: conductor-like screening model for real solvents and Hansen solubility parameters. The optimal HVED parameters were as follows: frequency 100 Hz, pulse width 400 ns, gap between electrodes 15 mm, liquid to solid ratio 50 mL/g, voltage 15 and 20 kV for argon, and 20 and 25 kV for nitrogen gas. Green solvents were used, water and ethanol (25% and 50%). The comparison was done with modified conventional extraction (CE) extracted by magnetic stirring and physicochemical analyses of obtained extracts were done. Results showed that HVED extracts in average 2.13-times higher total phenol content compared to CE. Furthermore, nitrogen, longer treatment time and higher voltage enhanced higher yields in HVED extraction. HVED was confirmed to have a high potential for extraction of BACs from rosemary. The computational stimulation methods were confirmed by experimental study, ethanol had higher potential of solubility of BACs and aromas from rosemary compared to water.

## 1. Introduction

Rosemary (*Rosmarinus officinalis* L.) is an autochthonous Mediterranean herb from *Lamiaceae* family. From ancient times, rosemary has been used as flavoring agent and for medicinal purposes due to its intense aromatic odor and health benefits [1]. The biological activity of rosemary is mostly related to the phenolic compounds, such as carnosol, carnosic acid, and rosmarinic acid [2], and volatile compounds from essential oil like α-pinene, camphor, eucalyptol, or 1,8-cineole [3]. Due to valuable bioactive compounds (BACs), rosemary possess antioxidant [2], anticancer [4], diuretic [5], antimicrobial [6], antiproliferative [7], anti-inflammatory [8], and anti-hyperglycemic properties [9]. Rosemary leaves, extracts, and essential oil have received recognition as generally recognized as safe (GRAS) for their intended use, from Food and Drug Administration [10] and according to Commission Directive 2010/67/EU and Commission Directive 2010/69/EU. Therefore, rosemary products can be a useful functional ingredient for the production of new functional foods [11].

Mostly used conventional extraction (CE) techniques are often associated with long extraction time, use of organic solvents in huge amounts and possible thermal degradation of thermosensitive compounds such as antioxidants [12]. For that reason, various innovative methods have been developed that are within the principles of green extraction processes. Apart from solvent extraction and steam distillation techniques, rosemary extracts have already been prepared by several green extraction methods: ultrasound-assisted extraction (UAE), microwave-assisted extraction (MAE) [13,14], supercritical fluid extraction (SFE) [15,16,17], and pressurized liquid extraction (PLE) [18,19,20]. For example, UAE was used for extraction of bioactive compounds from dried rosemary. Increased recovery of carnosic acid was obtained in ethanol, while rosmarinic acid was better extracted using methanol as a solvent. [21,22]. In a similar report, UAE of rosemary produced a three-fold increase in concentration of rosmarinic and carnosic acid when compared to the solid–liquid extraction [23]. In a work by Jacotet-Navaro et al., (2015), several extraction techniques were compared. Carnosic and ursolic acid extraction from rosemary was enhanced by ultrasound, while microwave extraction was more suitable for rosmarinic acid extraction. Both procedures were performed with reduced energy consumption and carbon emission when compared to heat reflux extraction. UAE was performed at 40 °C, while MAE was performed at 70–150 °C. The extraction yield increased with temperature but might have caused increased degradation and loss of volatile components, which makes thermal methods less suitable for extraction of aromatic compounds.

A green nonthermal extraction method that has not been investigated for extractions from rosemary is high voltage electrical discharge (HVED)—cold plasma treatment [24]. HVED extraction is a novel, eco-friendly extraction technique, that has been efficiently used in the extraction of BACs from various plant sources. Furthermore, as a nonthermal technology, extraction with HVED is performed in mild temperatures (usually at room temperature) and the temperature elevation after the extraction is low, so the thermal degradation of BACs is prevented [25,26]. The extraction of intracellular compounds by HVED is enhanced by the phenomenon of electrical breakdown in liquid that provokes cell structure damage and formation of pores (electroporation). The electrical breakdown is managed by the liquid ionization that is presented from the application of a high voltage between two electrodes with a gas flow. This leads to the liquid turbulence and intense mixing, emission of high-intensity ultraviolet light, generation of active radicals, production of shock waves and also a bubble cavitation [27].

Most conventional organic solvents are volatile, flammable, explosive, toxic, and have a negative environmental impact. For that reason, another principle of green extraction is directed towards use of alternative green solvents. In order to define green solvents, Gu and Jérôme (2013) proposed 12 criteria for green solvents that should be fulfilled related to availability, price, recyclability, grade, synthesis, toxicity, biodegradability, performance, stability, flammability, storage, and renewability. The solvent is considered greener when compared to conventional solvents that should be replaced with alternative solvent that fulfils at least some of the mentioned criteria [28]. Among green solvents, the water and agro- or bio-solvents play an important role for the replacement of organic solvents. Such solvents are derived from a renewable resource produced from biomass such as wood, starch, vegetable oils, or fruits. They have a high solvent power, are biodegradable, non-toxic, and non-flammable [29]. In a work by Barbieri et al., (2020), UAE of polyphenols from rosemary was performed in ethanol and natural deep eutectic solvents (NADES). High viscosity of NADES was reduced by the addition of 10% of water. The extraction efficiency was comparable to the results of ethanol extraction, while the antioxidant capacity of choline-based extracts was significantly improved [30].

It is important to choose an optimal solvent for the desired extraction, and to reduce solvent usage during experimentation. Traditional experimental procedures for solvent selection use high amount of solvents to choose which one is suitable for the extraction of compounds. For that reason, various computational simulations for solvent selection have been developed, such as Hansen solubility parameters (HSPs) and conductor-like screening model for real solvents (COSMO–RS). These models use theoretical predictions for assessing the solubility of targeted compounds in each solvent [31].

Since HVED treatment involves many processes, different reactive compounds are also formed. Beside generation of free radical species, there is also a possibility of electrodes abrasion and release of metals to the sample. For that reason, it is important to monitor preferred substances like phenols, antioxidants and volatile compounds, but also metals and other undesired compounds. Near infrared (NIR) spectroscopy has been utilized widely in the food and agribusiness ventures in the course of the last 20–30 years to decide significant parts in numerous agrarian items and plant materials [32,33,34]. In regard to determination of qualitative and quantitative characteristics of agricultural and food products, NIR offers a number of advantages over traditional analytical methods: it is a fast, physical, non-destructive, and non-invasive method, requires minimal or no sample preparation, no reagents are required, and no hazardous wastes are produced [35]. The wavelength range of the NIR spectroscopy from 750 to 2500 nm [36] is related to the vibration of molecules, especially the bands that are due to hydrogen (C-H, O-H, and N-H) vibrations [37,38]. But although the spectra recording is simple, user friendly (no additional sample preparation and use of chemicals) the interpretation of NIR spectra is very complex and chemometric methods are required to extract relevant information and reduce those that are less informative. The most common used tool is principal component analysis (PCA), quantitative analysis using multivariate calibration methods and qualitative analysis using multivariate classification techniques [34,38]. 

The aim of this work was to understand the green extraction of BACs and volatile compounds from rosemary using computational programs (HSPs and COSMO–RS) and experimental extractions. The experimental solvent extraction was carried out using HVED as a nonthermal technology. The obtained extracts were analyzed for physical and chemical (analytical) parameters. Furthermore, analyses of pesticides and metals in dry rosemary leaves and in HVED extracts were also performed.

## 2. Results

**Figure 1:**
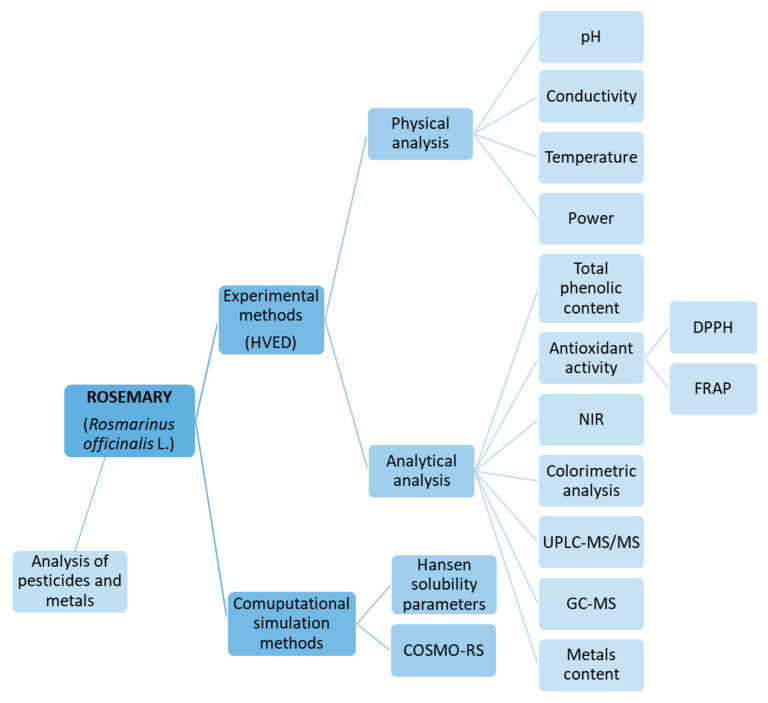
Flowchart of performed analysis in the paper.

The extraction of BACs and volatile compounds from dried rosemary leaves were assessed by computational simulation methods and experimental analysis using HVED. Computational simulation methods were performed by HSPs and COSMO–RS where various green solvents were assessed in comparison with conventional solvent n-hexane in order to theoretically predict the probability of solution of BACs from rosemary. Experimental extraction method from rosemary was done using water and ethanol solutions (25 and 50%) as green solvents by means of HVED, and it was compared with modified CE under the same extraction conditions. The obtained extracts by CE and HVED were analyzed for physical parameters (pH, conductivity, temperature, and power), non-volatile compounds (total phenolic content (TPC), antioxidant capacity by 2-diphenyl-2-picrylhydrazyl (DPPH) free radical assay and ferric reducing antioxidant power (FRAP) method, NIR and ultra-performance liquid chromatography-tandem mass spectrometry (UPLC–MS/MS)), volatile compounds by headspace solid-phase microextraction/gas chromatography-mass spectrometry (HS–SPME/GC–MS), and metal content. Additionally, analyzes of pesticides and metals in dry rosemary leaves were performed to assess the safety of raw material for further processing and human consumption. The flowchart of all performed analysis from rosemary is presented in Figure 1. 

### 2.1. Computational Simulation Methods for Assessing Solubility of Rosemary Compounds

The solubility parameters of green solvents for extraction of BACs and aromas from rosemary leaves have been studied by means of the HSP and COSMO–RS theoretical predictions. For both models, solubility results have been presented for various green solvents (ethyl acetate, methylacetate, ethyl oleate, ethanol, 1-butanol, isopropanol, methanol, limonene, α-pinene, cymene, β-myrcene, cyclopentyl methyl ether (CPME), dimethyl carbonate, methyltetrahydrofuran (MeTHF), and water) compared to conventional *n*-hexane (first column). The HSPs for rosemary compounds were assessed at room temperature (20 °C) for different solvents and relative energy difference (RED) values are summarized in Table 1. RED results have been used to quantify the solutes–solvents interaction.

COSMO–RS combines quantum chemical considerations (COSMO) and statistical thermodynamics (RS) to determine and predict thermodynamic properties without experimental data. The computational simulation results derived by COSMO–RS for rosemary compounds at room temperature is presented in Table 2.

### 2.2. Experimental Method for Extraction of Bioactive Compounds and Aromas from Rosemary by High Voltage Electrical Discharges

Experimental method for assessing the solubility of BACs from rosemary was performed by green extraction method using HVED and green solvents water and ethanol (25 and 50%). For comparison of results, a modified CE by magnetic stirring was performed at same conditions as HVED: at room temperature, extraction times 3 and 9 min, ratio plant:solvent 1 g:50 mL. For all results in experimental procedure, “R” stands for rosemary, “N” for nitrogen, and “A” for argon. For HVED, numbers 1–12 show the order of conducted treatment. For CE treatments, 3 and 9 are referred to extraction time while 0, 25, and 50 stands for concentration of an ethanol solvent (%).

Since the aim of the extraction with HVED was to achieve electrical discharge that is responsible for plant cell disruption and consequently the extraction of BACs from intracellular area. During the experiments, it was difficult to achieve discharge using nitrogen under 20 kV, therefore, for nitrogen treatments voltage of 20 and 25 kV were chosen, while lower voltages of 15 and 20 kV were obtained with argon treatments. All results were measured in duplicates and are presented as average ± standard deviation (SD).

The experiment design performed in STATGRAPHICS Centurion software is presented in Table 3.

#### 2.2.1. Determination of Physical Parameters of Rosemary Extracts

**Figure 2:**
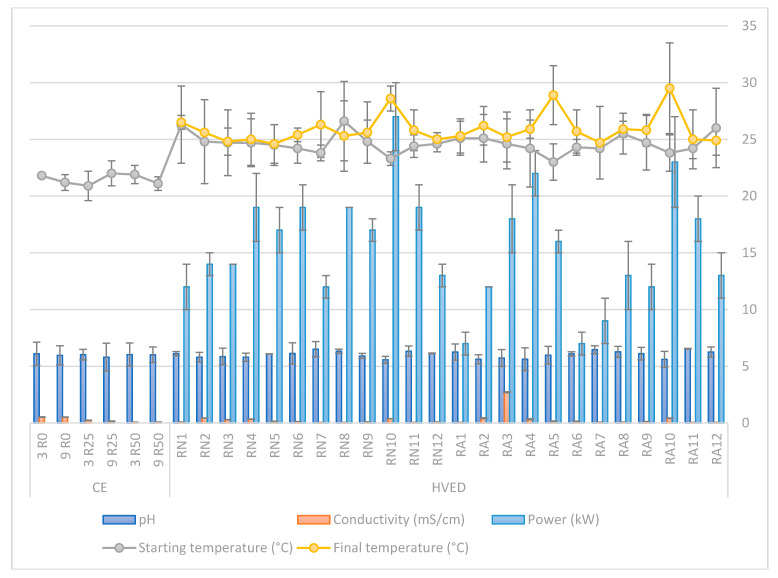
Values of pH, conductivity (mS/cm) for CE and HVED treated samples, starting temperature (°C), final temperature (°C) and power (kW) after HVED treatments.

Results of physical parameters of CE and HVED treatment are given Figure 2, including pH, conductivity, power and temperature before and after the HVED treatment.

#### 2.2.2. Determination of Phenols and Antioxidant Activity of Rosemary Extracts

**Figure 3:**
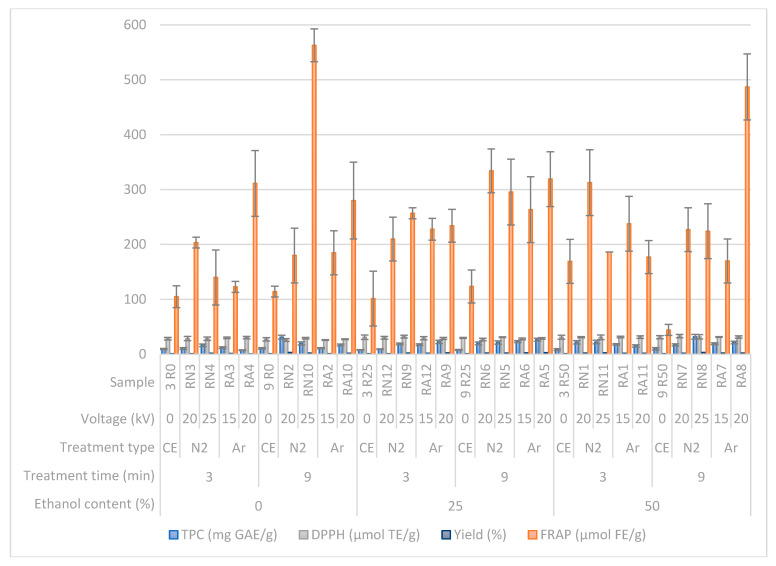
Determination of bioactive compounds—total phenolic compounds (TPC) values, antioxidant activity (2-diphenyl-2-picrylhydrazyl (DPPH) and ferric reducing ability of plasma (FRAP) and yield of extraction—measurements for CE and HVED treated samples. TPC = total phenolic content, DPPH = 2,2-diphenyl-2-picrylhydrazyl free radical assay, FRAP = ferric reducing ability of plasma; Treatment type: CE—conventional extraction, N_2_—HVED treatment with nitrogen, Ar—HVED treatment with argon.

The difference between CE and HVED extraction according to results of TPC, antioxidant parameters and extraction yields are presented in Figure 3. TPC results ranged from 7.21 to 31.64 mg GAE/g of sample. Antioxidant capacity was measured with two different methods—DPPH and FRAP. DPPH ranged from 25.85 to 32.92 µmol TE/g of sample, while FRAP ranged from 44.07 to 562.64 µmol FE/g of sample. Yield of extraction was calculated as g GAE/g of sample × 100 (%).

#### 2.2.3. Near Infrared Spectroscopy and Qualitative Modeling

**Figure 4:**
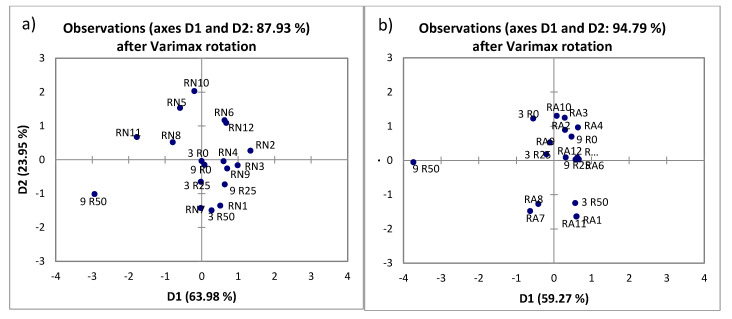
The principal component analysis (PCA) of rosemary extracts: (**a**) The data is denoting untreated rosemary samples, and rosemary samples treated with HVED using nitrogen; (**b**) The data is denoting untreated rosemary samples, and rosemary samples treated with HVED using argon.

The recorded NIR spectra in wavelength range from 904 to 1699 nm were used in the qualitative modelling using principal component analysis (PCA) to identify potential grouping (Figure 4) where the sample 9 R50 seemed to be an outlier. Additionally, the Grubbs test was conducted and this sample was confirmed as an outlier.

**Figure 5:**
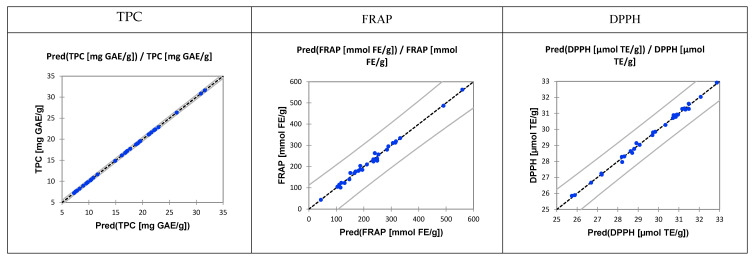
Control chart for predicted and experimental data of analyzed samples, using principal component regression (PCR) models.

Furthermore, modeling with different wavelength ranges was performed (Table S1) using the partial least squares regression (PLSR). Four different wavelength ranges were used for four models: Model 1: λ = 904–1699 nm; Model 2: λ = 1349–1699 nm; Model 3: λ = 904–932 and 1349–1699 nm; and Model 4: λ = 904–932 nm. Model efficacy was evaluated using the coefficient of determination (R^2^) and the regression point displacement that is the ratio of the standard error of performance (RPD) and the ratio of the range of reference chemistry values to standard error of prediction (RER). RPD is the ratio of standard deviation of the validation data set (SDv) and the standard error of prediction (SEP). How efficient this quantitative prediction of TPC, FRAP, and DPPH is on the rest of 40 % of the samples is presented in Figure 5.

#### 2.2.4. Determination of Color of Rosemary Extracts

The colorimetric analyses of rosemary extracts were measured under International Commission on Illumination (CIE)—L*a*b* color system. Results are shown in Table 4. Results of ∆C, ∆E, and ∆H are presented for HVED extracts as a result of total color difference, difference in tone color and difference in saturation, respectively, compared to same conditions with CE.

#### 2.2.5. Principal Component Analysis (PCA) of Rosemary Extracts

**Figure 6:**
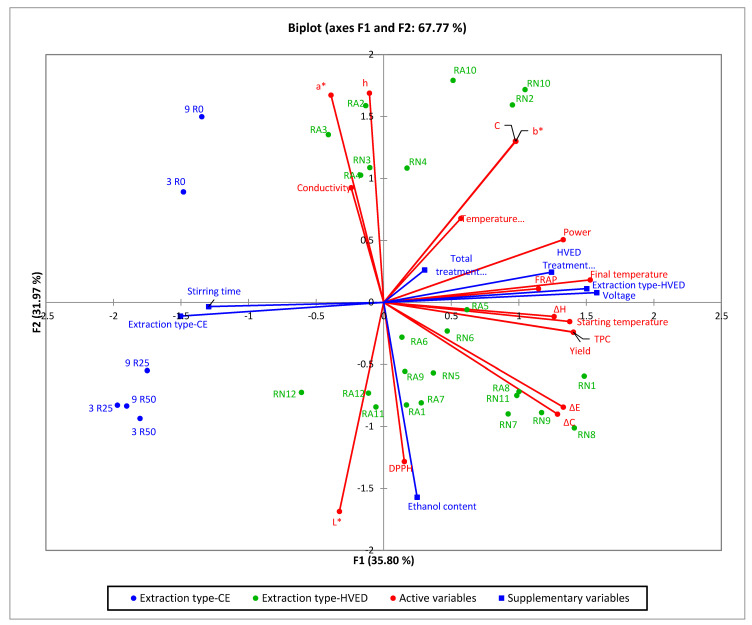
Biplot of the principal component analysis applied on all samples including the active variables (physical parameters, phenols and antioxidants, and parameters of color) and parameters of the experiment design as supplementary variables.

Data matrix used to identify similarities and/or differences in the data set presenting the samples as well as the physical and chemical properties. The PCAs ability to reduce the dimensionality and increasing the interpretability with minimal information lost was used on the data matrix of experimental data. The matrix included as active variables the physical parameters, total phenolic content and antioxidant capacity, as well as the parameters of color, while the supplementary data set were the experimental conditions (extraction type, HVED treatment time, stirring time, treatment time, voltage, and the ethanol content). The Extraction type (CE or HVED) were included in the analysis as qualitative variables (Figure 6). To present the differences in the samples which are extracted by CE or HVED, in the Supplementary Information are added boxplots for the TPC and AOX by use of DPPH and FRAP method and the yield, as well as the PCA biplot for different extractions and the physical-chemical properties Figures S3 and S4).

Modeling that has followed included the steps of calibration, validation and prediction. In the modelling were included the NIR spectra what is in detail explained in the Section 4.5. Calibration model was developed by principal component regression (PCR). From the data matrix 2/4 of it was used for the calibration and 1/4 was used for the validation while the remaining ¼ was used for the prediction. Validation was done by K-fold cross-validation.

#### 2.2.6. Ultra-Performance Liquid Chromatography–Tandem Mass Spectrometry (UPLC–MS/MS) Analysis of Phenolic Compounds from Rosemary Extracts

UPLC–MS/MS analysis was performed to quantify individual phenolic compounds (apigenin, carnosol, diosmetin, hydroxytyrosol, luteolin, oleanolic acid, quercetin, rosmarinic acid, p-cymene, camphor, thymol, and carvacrol) from rosemary extracts (Table 5).

#### 2.2.7. Determination of Volatile Compounds from Rosemary Extracts

Determination of main volatile compounds from rosemary extracts was performed by HS–SPME/GC–MS and analysis included eucalyptol, camphor, borneol and linalool. Results of performed analysis are presented in Table 6.

#### 2.2.8. Determination of Pesticides and Metals

Results of pesticides and metals measured from dried rosemary leaves are shown in Table 7. Also, table includes results of heavy metals measured in selected rosemary extracts. Maximum residue levels (MRLs) are given according to European Commission (EC) Regulations EC No. 396/2005 for pesticides for a rosemary plant and No. 1881/200 for metals for food supplements with rosemary, since rosemary is not listed in this Regulations as a plant.

## 3. Discussion

The aim of the present study was to provide the potential of green solvents to extract BACs from rosemary leaves comparing theoretical and experimental methods.

### 3.1. Computational Simulation Methods for Assessing Solubility of Rosemary Compounds

For extractions of BACs, *n*-hexane is one of the most used solvents due to its low polarity, optimal boiling point, easy removal from the product by evaporation and stability. On the other side, n-hexane is a solvent of petrochemical origin, which are nowadays strictly regulated by European Directives. For that reason, industries are forced to replace such solvents with more sustainable alternative solvents [39]. Therefore, various green solvents have been chosen for assessing the potential for extraction of BACs from rosemary (Table 1 and Table 2). Every solvent showed different theoretical solubility for rosemary BACs, and that can be explained by the differences in the solvent polarities. Generally, the optimum HSPs for very good solubility of solutes in solvents are presented with green color (Table 1). It can be concluded that many alternative (green) solvents are capable for extraction of BACs from rosemary, some even with higher affinity for extraction, compared to conventionally used *n*-hexane. According to RED results, by evaluating compounds with most green color (very good solubility), followed by yellow (medium solubility) and red color (poor solubility), the potential for extractions of BACs from rosemary was in the following order: CPME > limonene > cymene > MeTHF > ethyl oleate > β-myrcene > α-pinene > ethyl acetate > *n*-hexane > methylacetate > dimethyl carbonate > isopropanol > 1-butanol > methanol > ethanol > water. Results showed that α-pinene, cymene, β-myrcene, CPME, and ethyl oleate had high potential for extraction of volatile compounds such as monoterpenes and sesquiterpenes. For that reason, these solvents should be used for extraction of volatile compounds of essential oil from rosemary. Moreover, water and ethanol showed low potential for extraction of most evaluated compounds from rosemary.

Results of COSMO–RS solubility assessment (Table 2) showed similar trend for solvents like HSPs, although some differences have been noticed. COSMO–RS results showed following order according to most results with high probability of solubility (green color): MeTHF > CPME > ethyl acetate > methylacetate > 1-butanol > isopropanol > ethanol > ethyl-oleate > dimethylcarbonate > methanol > limonene > cymene > β-myrcene > α-pinene > *n*-hexane > water. It is clear that all green solvents have higher potential for extraction of rosemary compounds compared to conventional n-hexane, except water. According to results, ethyl acetate, methylacetate, ethanol, 1-butanol, isopropanol, methanol, CPME, dimethylcarbonate and MeTHF, showed high probability of solubility for diterpenes, triterpenes and flavonoids, which was not showed with HSPs assessment. 

### 3.2. Experimental Analysis of Extraction of BACs and Aromas from Rosemary Using HVED

#### 3.2.1. Physical Parameters of Rosemary Extracts

Results of pH, conductivity, power and temperature during extraction of rosemary BACs using HVED and CE are given in Figure 2. Temperature was measured before and after the HVED treatment and the maximum elevation of 5.9 °C was noted with sample RA5. In average, extracts treated for 9 min had 1 °C higher final temperature compared to extracts treated for 3 min. Also, maximum temperature was 29.5 °C after HVED treatment and it can be concluded that no significant elevation in temperature was noted during HVED treatment. Since all temperatures were under 30 °C, HVED was confirmed as a non-thermal extraction method. Results of pH for all extracts variated between 5.57 and 6.54 and no significant changes in pH were observed during the HVED treatment. pH and conductivity significantly depended (*p* ≤ 0.05) only on ethanol content. With higher content of ethanol in the solution, pH increased and conductivity decreased for both extraction types that is expected according to literature data [40]. 

The power used for the treatment with HVED changed from 7.0 to 27.0 kW. The highest power was noted for sample RN10 (27.0 ± 3.0 kW) which is expected since it was the sample treated for longer time 9 min) and the highest voltage (25 kV). Accordingly, power significantly increased with higher voltage used for the treatment and decreased with higher ethanol content in the sample.

#### 3.2.2. Total Phenolic Content and Antioxidant Activity of Rosemary Extracts

The goal of the extraction with HVED was to extract highest yield of phenolic compounds and antioxidants and compare results with same conditions by modified CE (at room temperature) (Figure 3). HVED treated samples showed 0.76–3.39-times higher yield of phenolic content for same extraction parameters than CE. On average, HVED had 2.13-times better yield of extraction of phenols than CE. The highest TPC value was noted for sample RN8 that was treated with HVED for 9 min at 25 kV with 50% of ethanol as a solvent. Nitrogen, longer treatment time and higher voltage yielded higher results of phenolic compounds, but only the treatment time had a statistically significant influence to TPC score (*p* ≤ 0.05). Ethanol content showed different trend for each treatment, with CE, the highest scores were obtained with water, HVED treatment with nitrogen was highest with 50% of ethanol, while treatment with argon had highest yield of TPC with 25% of ethanol. Bellumori et al., (2016) performed extractions from dried rosemary leaves by ultrasound-assisted extraction and microwave-assisted extraction for 10 min. Their results also showed higher amounts of TPC with ethanol as a solvent, compared to water, but their maximum obtained results were slightly higher compared to HVED, 35.0 mg/g for ultrasound and 36.6 mg/g DL for microwave-assisted extraction with ethanol (ACS grade, ≥99%), but different method for calculation was used [23]. 

Results of DPPH did not vary notably between extracts. However, the significant correlation was observed for DPPH with treatment time and ethanol content, it was higher with shorter treatment time and higher percentage of ethanol in the solution. Some differences in DPPH results could be due to generation of free radicals that is characteristic for HVED treatment that could influence to the antioxidant activity of extracts and have a possibility to interact with DPPH radical. FRAP results showed similarities with TPC values for HVED extraction, it was higher with longer treatment time, higher voltage used and with treatment using nitrogen, compared to argon. In average, HVED extraction showed 2.39-times higher antioxidant capacity, according to FRAP results, when compared with CE. 

In total, higher results for phenolic content and antioxidants was noted with ethanol than with water. These results are in line with theoretical predictions with HSPs and COSMO–RS, although pure ethanol was used for calculations, while in experimental procedure 25 and 50% ethanol was used. Additionally, other green solvents were used for experimental assessment for extraction of BACs using HVED including limonene, α-pinene, glycerol, ethyl acetate and dimethyl carbonate. However, no electrical discharge was achieved during the extraction with HVED when mentioned solvents were used. This could be explained by high viscosity and density of these solvents [41,42]. Also, terpenes, such as limonene and pinene are oil solvents that are not able to mix with water and are therefore suitable for extraction of volatile compounds and not water-soluble compounds [41].

#### 3.2.3. Near Infrared Spectroscopy of Rosemary Extracts and Modeling

The recorded NIR spectra showed grouping in the range of λ = 904–1699 nm which is the result of different vibrations of molecules as C–H, O–H, and N–H bonds. NIR spectra was specific in two regions: 904–925 nm and 1350–1699 nm. Overlapping of NIR scans is visible with specific differences in the range of 904–925 nm, indicating absorption detecting differences of the third overtone region and detecting different vibration of C–H bonds as well as HOH region at 1400 nm (water λ = 1400–1460 nm) and continues to the end of the recorded spectra indicating differences in the vibrations of the first overtone of C–H3, Ar–CH, C–H, and C–H2 bonds and second overtone of O–H, N–H, Ar–CH, and R–OH [43,44].

From those findings it was clear that the content of TPC and the antioxidative activity could be predicted. A part of the input spectral data was used for the model training (60%). After the training followed the PLSR model evaluation and its testing on unknown samples. Our aim was to predict those parameters not only qualitative but also quantitatively. In order to gain more accurate models, four different wavelength ranges were used (Model 1: λ = 904–1699 nm; Model 2: λ = 1349–1699 nm; Model 3: λ = 904–932 and 1349–1699 nm; and Model 4: λ = 904–932 nm). The first model (Table S1) which included the total range of NIR spectra resulted with the best model efficiency parameters (RPDs > 3 and RER > 10) what is an indication of very good quantitative model prediction [45]. The efficacy of model for prediction of TPC, DPPH, and FRAP using NIR is presented in Figure 5.

#### 3.2.4. Colorimetric Analysis of Rosemary Extracts

The color of the product is an important aspect that specifies the commercial quality of the product and has an effect to the consumer’s final purchase decision [46]. The color measurements of the different extracts prepared by conventional and HVED extraction are given in terms of L*, a*, b*, C, and h values under CIE—L*a*b* color system. According to the results, HVED treated samples had lower values of L* (darker), a* (more green), and higher values of parameter b* (more yellow) when compared to CE in same conditions (Table 4). The HVED extraction also caused increasing in parameter C resulting with increase and discernible difference in color intensity and slight increase in hue of extracts. Differences with untreated extracts are expressed as ΔE—total color difference, ΔC—difference in tone color; ΔH—saturation. Based on these data with more intense coloring of HVED extracts (mass and the solvent used were equally as with CE), it can be concluded that HVED treatment had damaged the cell structure and that the pigments contained within the plant cells exited the cell surface causing changes in extract color [47]. Since the cavitation is caused, the process of extraction of BACs is also facilitated [48]. In our study, ethanol content had significant effect on color parameters (L*, a* and b*) in rosemary HVED treated samples with both argon and nitrogen. All colorimetric parameters, except ΔH, had statistically significant dependence (*p* ≤ 0.05) on ethanol content—with higher ethanol content, parameters L, ΔC, and ΔE increased, while a*, b*, C, and h decreased. Saturation significantly depended on treatment time and gas used, more saturation was noted with longer treatment time and use of nitrogen during the treatment. 

In general, results indicated that HVED extracts had dark greenish-brown color with darker color when compared to CE which is associated also with higher phenolic compounds and antioxidant content. Plasma reactive species induce release of some BACs that are covalently bonded to the plant matrix which accordingly results with increase in TPC and greater antioxidant capacity and consequently with changes in extract colors. The reason is that phenolic compounds result with different color in free and bound forms [49,50].

#### 3.2.5. Principal Component Analysis (PCA) of Rosemary Extracts

Based on the PCA biplot showed in Figure 6, the extraction type divided samples on the left and right part of chart quadrants. Extraction with CE positioned the samples in the second and third quadrant while the HVED extraction has spread the samples in all four quadrants with the main sample concentration in the fourth quadrant. The physical composition, content of TPC and antioxidant capacity as well the color parameters of the extracts. The experiment conditions are dominantly positioned near to the first principal component, PC1 (time (of HVED treatment, stirring and total treatment), voltage) with the exception—ethanol content.

The subjected table to the PCA chart-squared cosines of the variables asserts the HVED treatment time, stirring time, total treatment time and voltage used as experiment condition as values for which the squared cosine is the largest. 

Biplot in the form where the active variables (physical properties, TPC, antioxidant capacity (DPPH and FRAP), and color parameters) are related to the supplementary which are the experimental design parameters, show the correlations between them and for which samples are they dominant. So accordingly, in the fourth quadrant the almost overlap of parameters as the DPPH content and the ethanol content indicating that the antioxidant capacity by DPPH method was higher in those samples where the ethanol content was 25% or 50% (Samples RA1; RA7; RA8; and all other positioned in the fourth quadrant, with the higher DPPH values of 31.28% (RA1 and RA7) and 31.31% (RA8), respectively. Applying the same rule of data relation analysis in the third quadrant, samples RN12; RA11; and RA12 as well as 3 R25; 3 R50; 9 R25; and 9 R50 will have the highest values of the parameter L*, what confirmed the results presented in Table 4.

#### 3.2.6. Analysis of Individual Bioactive Compounds from Rosemary Extracts

Data of UPLC–MS/MS analysis for individual phenolic compounds (Table 5) showed that main phenolic compounds in rosemary are: apigenin, diosmetin and rosmarinic acid. Comparison of phenolic compound in different type of extraction (CE and HVED) and conditions in extraction type showed that: apigenin, carnosol, diosmetin, hydroxytyrosol, luteolin, oleanolic acid, oleuropein, quercetin, and rosmarinic acid were higher in HVED extracts compared with CE extracts. The example of chromatograms for selected extracts is presented in Supplementary Materials (Figure S1). For this purpose, the extract with highest content of phenolic compounds detected with UPLC–MS/MS was chosen (RN9, Figure S1b) and compared with extract extracted with CE (3 R25, Figure S1a) under same conditions (3 min, 25 % of ethanol).

Statistical analysis showed that most of the measured compounds significantly depended only on ethanol content (apigenin, carnosol, diosmetin, hydroxytyrosol, luteolin, oleanolic acid, oleuropein, quercetin, and rosmarinic acid) and additionally, apigenin, carnosol, diosmetin, hydroxytyrosol, and luteolin depended on treatment time.

HVED is considered to be energy- and cost- saving method for successful extraction of phenolic compounds from rosemary. However, further analysis of energy and environmental impact should be performed. Hirondart et al., (2020) have obtained rosmarinic acid, carnosic acid, and carnosol by PLE in hydroalcoholic solution and conventional Soxhlet extraction. Extract yields of bioactive compounds were similar with both methods, energy consumption was lower for PLE extraction because less solvent had to be heated, and the cost was reduced with a smaller amount of waste generated [51].

When compared with theoretical results, it is clear that a similar trend for solution in water and ethanol was noticed. With higher ethanol content, solubility of most of extracted compounds increased, as well as a sum of all phenolic compounds. According to HSP results, all compounds that were analyzed with UPLC–MS/MS showed poor solubility (red color in Table 1) in both water end ethanol, but better results (higher solubility) was presented with ethanol as a solvent. However, COSMO–RS results gave better solubility results for extraction with ethanol. Results showed that carnosol, rosmarinic acid, apigenin and diosmetin have 100% of solubility in ethanol (green color in Table 2), while camphor has 44.06% solubility (yellow color). Experimental results confirmed these results since apigenin and carnosol showed the highest results with 50% of ethanol as a solvent, 164.68 and 349.80 ng/mL respectively, and diosmetin and rosmarinic acid showed highest measured results 25% of ethanol as an extraction solvent, 376.44 and 6002.35 ng/mL, respectively. Camphor was found in small amounts in all extracts (<1 ng/mL) except in water extract RA2 (1.78 ng/mL). Results are in line with one previously reported for oregano [52].

#### 3.2.7. Analysis of Volatile Compounds from Rosemary Extracts

HS–SPME is a rapid, simple, inexpensive, solvent-free and highly sensitive technique [53]. Volatile organic compounds composition is strongly dependent on the extraction method. Results of the chemical composition of HS is presented in Table 6. The predominant HS compound was the cyclic monoterpene ether eucalyptol (40.33–2.28%). A second compound was cyclic monoterpene ketone camphor (26.70–0.99%), followed by bicyclic monoterpene borneol (15.99–0.24%). Terpene alcohol linalool (3.46–1.19%) was found in a smaller percentage. The concentration of these terpenes depends on the treatment of the plant with ethanol content or gas used (nitrogen or argon). From Table 6, it is notable that when water is used as an extraction solvent, the percentage of all three monoterpenes is high except for linalool. It was difficult to characterize volatile compounds in extracts with ethanol since overlapping profile of peaks happened with ethanol peak. Therefore, no results for most samples with ethanol were presented. The traceability is similar in the RN2-RN6 sample as in the RA2-RA6 sample, except that the concentration of linalool monoterpene alcohol in these samples deviated. Linalool was not found in the RN10 sample, while it was found in RA10. Chromatograms for two extracts with highest concentrations obtained by CE (3 R0, Figure S2a) and HVED (RA10, Figure S2b) are given in Supplementary Materials.

It was not possible to compare data with theoretical predictions for ethanol and water solubility of volatile compounds, since incomplete results are provided for ethanol extracts. However, the comparison between camphor and borneol can be provided to compare theoretical and experimental data. HSPs showed poor solubility in both ethanol and water for both compounds, but borneol had slightly higher chances for solubility (RED = 3.33 in ethanol and 9.3 in water), compared to camphor (RED = 4.49 for ethanol and 10.45 for water). From COSMO–RS results, it was also predicted that camphor and borneol have low chances for solubility in water, 0.1% and 0.02%, respectively, but different results were given for ethanol: camphor has medium probability of solubility (44.06%), while borneol has high probability (85.11%). Experimental results were opposite and higher concentrations of camphor were found in all extracts, compared to borneol. Moreover, results were closer to COSMO–RS results than HSPs since both compounds were extracted in higher concentrations: 37.83% of camphor was extracted in sample RA10 and 15.99% of borneol in sample RN10. Mena et al., (2016) showed similar results in conventionally performed acetone-based extraction from rosemary, they have extracted more camphor (41.52 ± 6.00 µg/g) than borneol (11.92 ± 2.01 µg/g) in their extracts [54]. 

SFE is a procedure recently used for the extraction of bioactive compounds and purification of rosemary essential oil. Mouahid et al., (2017) have compared the efficiency of SFE and hydrodistillation of rosemary leaves. The essential oil obtained by SFE had an increase in yield of monoterpenoids for 37%, while yields of individual monoterpenes varied [16]. Pereira et al., (2007) have performed a cost analysis of the extraction of rosemary essential oil by SFE and steam distillation. The manufacturing cost with SFE was lowered, while the lower profitability of steam distillation was a consequence of higher energy consumption and lower content of essential oil in the extracts [55]. SFE was used to extract the volatiles from rosemary and combined with SWE for the recovery of polyphenols from the produced extract. A combination of these processes resulted in a 28% reduction of operating cost when compared to the separate use of these techniques [56]. Since HVED was presented as a method that is more efficient for extraction of non-volatile than volatile compounds, it could be considered to be used in a combination with some other techniques for better extraction efficiency as well.

#### 3.2.8. Analysis of Pesticides and Metals

Although rosemary could be considered as a nutritional supplement, there are still no categories in European Commission (EC) Regulation, herbs or plant tea, therefore the high levels of pesticides and heavy metals that can be found in its dried leaves could possess serious toxicological effects on human health. For that reason, the analysis of pesticides and heavy metals in the rosemary samples were measured and are presented in Table 7. This analysis is important for preparing healthy extracts from dried rosemary leaves that could be further used for new functional food. Trace analysis of pesticides residues were analyzed in dried leaves and for heavy metals residues were analyzed in dried leaves and HVED extracts. Residues levels of all pesticides were lower than limit of quantitation of method which is quite below maximum residue level (MRL) according to EC Regulations No. 396/2005 for rosemary as a plant. In EC regulation MRL of some pesticides with similar structures (such as DDT, endosulfan, aldrin, heptachlor, etc.) are grouped. 

Residue levels of Lead (Pb) and Cadmium (Cd) in dried rosemary leaves were also below the limit of quantitation of the method, and only the level of Mercury (Hg) was slightly higher than limit of quantitation. However, all these values were quite below MRL according to EC No. 1881/2006. Furthermore, other metals (Chromium (Cr), Nickel (Ni), Manganese (Mn), Iron (Fe), Copper (Cu), and Zinc (Zn)) were measured in selected HVED extracts with high phenolic and antioxidant content (RA8, RN7, RN9, and RN11) as well and results are presented in Table 7. These metals are not included in EC Regulations so no MRL data were provided. Although the data should not be compared since data before the HVED extraction are given per g of dried herb and data after HVED extraction are given per gram of extract, it is clear that content of Cr and Ni increased in extracts, while level of other metals decreased after HVED extraction. From this data it is notable that during the HVED treatment, levels of some toxic metals are increasing that could be the result of abrasion of electrodes during the treatment.

Rosemary plant was compliant regarding content of contaminants, pesticide residues and toxic metals. With respect to this, obtained results showed that rosemary samples are safe for use in human dietary. On the other hand, some changes in levels of metals could happen during the treatment and further detailed analyses should be done to assess this issue.

## 4. Materials and Methods

The concept of this work is presented in Figure 1, where all analysis performed for rosemary are presented as a flowchart.

### 4.1. Plant Materials

Dried rosemary leaves (*Rosmarinus officinalis* L.) were provided by local drugstore (Suban d.o.o., Samobor). Herbs were stored in polyethylene bags in a dark and dry place until extractions. Dried rosemary leaves were grinded to plant particle size distribution of d(0.1) ≤ 3 9.683 μm; d(0.5) ≤ 224.816 μm; d(0.9) ≤ 425.819 μm measured by the laser particle size analyzer Mastersizer 2000 (Malvern Instruments GmbH, Herrenberg, Germany). For the extraction, 1 g of herb material was weighted into the beaker of 100 mL and mixed with 50 mL of extracting solvent at room temperature (22 °C). Extraction was carried out using distilled water, 25% and 50% aqueous ethanol (*v*/*v*) as extraction solvents.

### 4.2. Computational Simulation Methods

#### 4.2.1. Hansen Solubility Parameters (HSPs)

HSP provide a convenient and efficient way for characterization of solute-solvent interactions according to the classical “like dissolves like” rule. A detailed concept of HSPs is described in Aissou et al., (2017) [57]. For solvent optimization, a simple composite affinity parameter, the RED number, has been calculated to determine the solubility between solvents and solutes.

(1) $$\mathrm{RED}=\frac{\mathrm{Ra}}{\mathrm{Ro}}$$

where R_o_ is the radius of a Hansen solubility sphere and R_a_ is the distance of a solvent from the center of the Hansen solubility sphere. 

A potentially good solvent has RED number smaller than 1 (the compound has similar properties and will dissolve), while medium and poor solvents have RED values of from one to three and more than 3, respectively. The chemical structures of the solvents and solutes discussed in this article could be mutually transformed by JChemPaint version 3.3 (GitHub Pages, San Francisco, CA, USA) to their simplified molecular input line entry syntax (SMILES) notations, which were subsequently used to calculate the solubility parameters of the solvents and compounds (HSPiP Version 4.0, Hansen Solubility, Hørsholm, Denmark).

#### 4.2.2. COSMO–RS Software

The COSMO–RS was developed by Klamt and co-workers as a statistical thermodynamic method for molecular description and solvent screening based on a quantum-chemical approach [58]. COSMO–RS prediction is a two-step procedure—microscopic and macroscopic. The procedure was explained in details by Aissou et al., (2017) [57]. The COSMOthermX program (version C30 release 13.01, COSMOlogic, Leverkusen, Germany) was used to calculate the relative solubility between the solid compound and the liquid solvent in terms of the logarithm of the solubility in mole fractions (log_10_(x_solub_)). The logarithm of the best solubility was set to 0 and all other solvents were given relative to the best solvent. Also, the logarithm was transformed into probability of solubility (%). The calculation was performed at room temperature (20 °C) and at boiling temperature for each solvent.

### 4.3. High Voltage Electrical Discharge (HVED) and Conventional Extraction (CE)

**Figure 7:**
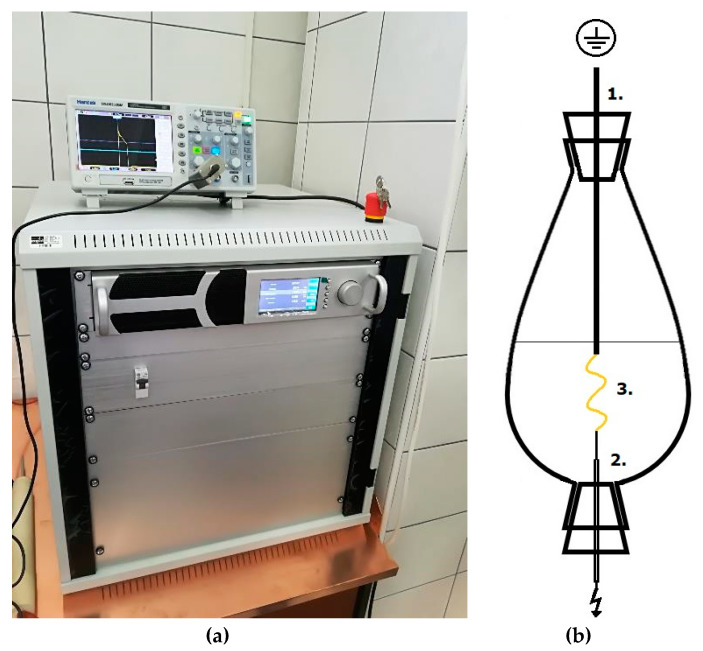
Set-up of generator and reactor for HVED treatments: (**a**) HVED and plasma generator „IMP-SSPG-1200” (Impel group d.o.o., Zagreb, Croatia); (**b**) Beaker shaped reactor: (1)—ground electrode; (2)—high voltage electrode (needle with empty interior for argon and nitrogen flow) during treatments; and (3)—discharge (plasma).

HVED was performed with “IMP-SSPG-1200” generator (Impel group d.o.o., Zagreb, Croatia) that generated rectangular pulses using direct current and achieving high voltage. Maximum adjustable current was 30 mA and voltage up to 25 kV. Based on conducted preliminary experiments with different HVED parameters (frequency, voltage, pulse length, distance between electrodes, and ratio mass to solvent), fixed HVED parameters were chosen as follows: frequency of 100 Hz, pulse width 0.4 microseconds, voltage 15 and 20 kV for argon gas, and 20 and 25 kV for nitrogen gas, the gap between electrodes of 15 mm, treatment duration 3 and 9 min, and ratio mass to solvent 1 g:50 mL (according to pharmacopoeia). Mixture of herb material and solvent was transferred to beaker shaped reactor of 100 mL. This reactor that is opened on both sides was fitted with silicone tops with 1 cm in diameter. Silicone tops were used due to easier mounting of the electrode from the top and needle form the bottom. Gases (argon or nitrogen) were flowed in through the needle with the flow 0.5–1 L/min. Set-up of generator and reactor are shown in Figure 7. For measuring the output voltage (data not shown), oscilloscope Hantek DS05202BM (Tektronix, Inc., Beaverton, OR, USA) connected to the high voltage probe Tektronix P6015A (Hantek Electronic Co., Ltd., Qingdao, China) was used.

For comparison, modified CE (untreated samples) was performed at room temperature as well, by dissolving the dried rosemary material in the solvent with light magnetic stirring during 3 or 9 min. Both extractions, HVED and CE, were performed in duplicates.

### 4.4. Analytical Methods

#### 4.4.1. Determination of Total Phenolic Content (TPC)

For determination of TPC of rosemary extracts, a Folin–Ciocalteu method was used [59] with slight modifications. A volume of 0.1 mL of extract (appropriately diluted) was mixed with 0.2 mL of Folin–Ciocalteu reagent. After 3 min 1 mL of 20% Na_2_CO_3_ (*m*/*v*) was added. After thorough mixing by vortex, the reaction mixtures were incubated at 50 °C for 25 min, followed by absorbance reading at 765 nm against blank (instead of an extract, extraction solvent was used). The calibration curve was prepared using 50 to 500 mg/L of gallic acid in ethanol as a standard. The concentration of TPC was expressed in mg of gallic acid equivalents per gram of sample (mg GAE/g of sample).

#### 4.4.2. 2-Diphenyl-2-Picrylhydrazyl (DPPH) Free Radical Assay

The antioxidant activity of rosemary extracts determined by DPPH method was determined as reported by Shortle et al., (2014) [59] with slight modifications. An aliquot (0.75 mL) of rosemary extracts or methanol solution of Trolox (25–200 mM) was mixed with 1.5 mL of 0.5 mM DPPH methanolic solution. After mixing, the solutions were stored in the dark for 20min at room temperature and the absorbance was measured at 517 nm against 100% methanol as a blank. The results were calculated using calibration curve for Trolox and expressed as µmol of Trolox equivalents per gram of sample (µmol TE/g of sample).

#### 4.4.3. Ferric Reducing Antioxidant Power (FRAP) Assay 

The FRAP assay was conducted according to literature [59] with modifications. The FRAP reagent was prepared by mixing 0.3 M acetate buffer (pH 3.6) with 10 mM TPTZ solution and 20 mM FeCl_3_ solution in ratio 10:1:1. An aliquot (80 μL) of rosemary extract was mixed with 240 μL of water and 2080 μL of FRAP reagent. Following incubation at 37 °C for 5 min, the absorbance was measured at 595 nm. FRAP values were calculated according to the calibration curve for FeSO_4_·7H_2_O and expressed as μmol of Fe^2+^ equivalents (FE) per g of sample (μmol FE/g of sample).

#### 4.4.4. Near Infrared Spectroscopy (NIR) 

NIR spectroscopy was conducted using the NIR-128-1.7-USB/6.25/50μm (Control Development Inc., South Bend, IN, USA) to record sample spectra using the SPEC 32 Control Development software. NIR spectra was recorded in the wavelength range from 904 to 1699 nm. Each sample was recorded in triplicate and the average spectrum was calculated which was used for further processing.

#### 4.4.5. Colorimetric Evaluation of Rosemary Extracts

Color parameters for all trials was measured by Konica Minolta colorimeter CM 3500d (Konica Minolta, Tokyo, Japan) at CIE Standard Illuminant D65 by 8 mm thick plate. All measurements were conducted in the Specular Component Included (SCI) mode as previously reported [60]. The color measurements of the different extracts prepared by CE and HVED extraction are given in terms of L*, a*, b*, C, and h values under CIE—L*a*b* color system (L*—lightness from black to white; a*—from green to red, and b*—from blue to yellow; C—chroma; and h—hue angle). Differences compared to CE were expressed as ΔE—total color difference, ΔC—difference in tone color; and ΔH—saturation.

#### 4.4.6. Ultra-Performance Liquid Chromatography-Tandem Mass Spectrometry Characterization of Phenolic Compounds (UPLC–MS/MS)

UPLC–MS/MS Eksigent Expert Ultra LC 110, SCIEX 4500 QTRAP (SCIEX, USA) method for reference conditions [61] was conducted using Luna Omega 3 μm Polar C18 100 Å, 100 × 4.6 mm (column), thermostat column temperature 40 °C, automatic sampling temperature 4 °C, and injection volume of 10 μL. Mobile phases consisted of: A 100% H_2_O with 0.1% HCOOH (*v*/*v*) and B 100% acetonitrile with 0.1 % HCOOH (*v*/*v*) with mobile phase flow 0.40 mL/min. Gradient was set as follows: 1 min 10% B, 2 min 10% B, 15 min 90% B, 25 min 90% B, 27 min 10% B, 30 min 10% B. Determination conditions for MS/MS detector were: ionization -negative ionization mode atmospheric pressure (API)—negative ionization at atmospheric pressure; ionization temperature: 500 °C, i.e., gas temperature combining the mobile phase at the exit from the capillary before ionization. Voltage on the electrode after capillary and next to ionization (Ion Spray Voltage) was −4500 V.

#### 4.4.7. Headspace Solid-Phase Microextraction (HS–SPME) Followed by Gas Chromatography and Mass Spectrometry Analysis (GC–MS)

HS–SPME was performed with a manual SPME holder using three fiber covered with DVB/CAR/PDMS obtained from Supelco Co. (Bellefonte, PA, USA). For HS–SPME, the finely samples 2 mL were placed separately in 10 mL glass vials and hermetically sealed. The vials were maintained at 60 °C during equilibration (15 min) and extraction (45 min). Thereafter, the SPME fiber was withdrawn and inserted into GC–MS injector (250 °C) for 6 min for thermal desorption. The procedure was similar as in previous paper [62]. 

Gas chromatography and mass spectrometry (GC–MS) analyses were done on an Agilent Technologies (Palo Alto, CA, USA) gas chromatograph model 7890A equipped with a mass spectrometer (MSD) model 5977E (Palo Alto, CA, USA) and HP-5MS capillary column (5% phenyl-methylpolysiloxane, Agilent J & W). The GC conditions were the same as reported previously [62]. In brief, the oven temperature was set at 70 °C for 2 min, then increased from 70 to 200 °C (3 °C /min) and held at 200 °C for 18 min; the carrier gas was helium (1.0 mL/min). The compounds identification was based on the comparison of their retention indices (RI), determined relatively to the retention times of *n*-alkanes (C_9_–C_25_), with those reported in the literature [63] and those from Wiley 9 (Wiley, New York, NY, USA) and NIST 14 (National Institute of Standards and Technology; Gaithersburg, MD, USA) mass spectral database. The percentage composition of the samples was computed from the GC peak areas using the normalization method (without correction factors).

#### 4.4.8. Determination of Pesticides and Metals in Rosemary Samples

The contents of the pesticides were performed by modified procedures with following national regulations HRN EN ISO 12393-1, 12393-2, and 12393-3: 2013, i.e., extraction with petroleum ether/dichloromethane and determination using the GC-ECD Varian CP-3800 instrument (Varian, Inc., Walnut Creek, CA, USA). Metal trace content was determined according to the HRN EN ISO 14084: 2005 procedure, or by wet sample digestion by HNO_3_ (microwave digestion) with microwave reaction system Multiwave 3000 (Anton Paar GmbH, Graz, Austria). Determination of metals were conducted on the Perkin Elmer AAS Analyst 800 and ICP-MS Perkin Elmer NexION 300× (PerkinElmer, Inc., Waltham, MA, USA), while Hg traces were determined by the Leco AMA254 Hg analyzer (LECO Inc., St. Joseph, MI, USA).

### 4.5. Experimental Design and Statistical Analysis

The experiment was designed in STATGRAPHICS Centurion (StatPoint Technologies Inc., Warrenton, VA, USA) software. Multifactorial design consisting of 12 experimental trials using per gas (argon and nitrogen). The three chosen independent variables for HVED assisted extraction were: treatment time (3 and 9 min), voltage applied and gas type (15 or 20 kV for argon, and 20 or 25 kV for nitrogen) and concentration of ethanol (0%, 25%, or 50%). For CE, the independent variables included: concentration of ethanol (0%, 25%, or 50%) and treatment time (3 and 9 min). The experimental design is presented in Table 3. A total of 30 extracts were prepared in duplicates.

In order to provide information about experimental results, a descriptive statistic was used. Analysis of covariance (ANCOVA) was used for assessment of correlation and all parameters according to dependent variables (gas, treatment time, voltage, ethanol content). The *p*-values present the statistical significance of each of the factor and it was significant at *p* ≤ 0.05. Statistics was performed using XLStat (MS Excel 2010) (data not shown).

Information investigation of NIR spectra includes preprocessing and calibration modeling [64] where preprocessing will minimize commotions and undesirable components in spectra, which are subjected to construction of calibration models. Data matrix which included NIR spectra and physical-chemical properties of the samples consisted of 128 rows and 797 columns. This matrix was used for the identification of qualitative differences, by use of principal component analysis (PCA) as well as in the modelling that followed. The NIR spectra were pre-treated to enhance the prediction accuracy. Several spectra pre-treatment methods were arranged to the original absorbance spectra such as multiplicative scatter correction (MSC), standard normal variate (SNV), Smoothing (Moving Average, Gausian and Median filter, and Savitzky–Golay), first and second derivative absorbance (d1a and d2a), Savitzk–Golay first and second derivative absorbance (S-G d1a and S-G d2a), and the combination of the MSC and SNV + d1a or d2a, but as the most effective was the Savitzky–Golay first derivation (S-G d1a). Calibration model was developed by principal component regression (PCR). From the data matrix 2/4 of it was used for the calibration and 1/4 was used for the validation while the remaining ¼ was used for the prediction. Validation was done by K-fold cross-validation. [65]. Then followed the application of the multivariate tool mostly used in model prediction, the PLSR and PCR [45]. As in the case of pretreatments, one or more multivariate tools can be used in the calibration, which implies quantitative or qualitative analysis. Model efficacy is evaluated using R^2^ and the regression point displacement that is the ratio of the standard RPD and the ratio of the range of reference chemistry values to RER. RPD is the ratio of SDv and SEP, while RER range, min and max, and minimum and maximum values of the validation set. All data analyses were conducted in MS Excel and its additional statistical tool pack: XLStat.

## 5. Conclusions

In this study, the potential of high voltage discharges for green solvent extraction of BACs and aromas from rosemary leaves was assessed by computational simulation and experimental method by means of HVED. The experimental results were compared with untreated samples (modified CE) and HVED was presented to yield 2.13-times higher TPC and 2.39-times higher antioxidant capacity. Nitrogen, longer treatment time, and higher voltage yielded higher results of phenolic compounds and antioxidants. Also, NIR spectra and modelling with analytical data were shown as an extremely useful tools that can help in assessing whether there is a “cost-effectiveness” of extracting phenols or antioxidants from specific samples, in a quick and easy way. The results presented that NIR spectroscopy combined with chemometrics approach gave accurate TPC, FRAP, and DPPH content prediction, showing that indicates the potential of the method in estimating the quantitative expected antioxidant potential as well as the content of total phenols. Generally, HVED extracts had a dark greenish-brown color with darker color when compared to CE which is associated also with higher phenolic compounds and antioxidant content. An UPLC–MS/MS showed that main phenolic compounds in rosemary were apigenin, diosmetin, and rosmarinic acid, while the predominant volatile compounds in rosemary extracts was eucalyptol. Altogether, results showed that HVED confirmed high potential for extraction of BACs and food aromas from rosemary with increased yield of individual compounds and total phenolic and antioxidant properties, compared to untreated samples. Furthermore, rosemary was presented as safe raw material for further processing in human nutrition in terms of pesticides and metals. The computational stimulation methods were confirmed by experimental study, ethanol had higher potential of solubility of BACs and aromas from rosemary compared to water. Therefore, these theoretical prediction methods present a new approach for assessment of solubility of individual compounds in selected solvents that could impact to lower solvent usage during experimentation and lower environmental impact.

## Figures and Tables

**Table 1:** Hansen Solubility Parameter (HSP) values of relative energy difference (RED) of bioactive compounds from rosemary for different solvents.

	Compounds	n-Hexane	Ethyl Acetate	Methyl Acetate	Ethyl Oleate	Ethanol	1-Butanol	Isopro-panol	Methanol	Limonene	α-Pinene	Cymene	β-Myrcene	CPME	Dimethyl Carbonate	MeTHF	Water
Solvents																	
**Monoterpenes**																	
β-myrcene		1.04	1.45	2.77	0.1	4.5	3.39	3.56	5.57	0.67	0.32	0.67	0	0.5	2.36	0.91	10.49
p-cymen-7-ol		3.01	1.37	1.68	1.93	2.97	1.96	2.15	4.13	1.48	2.17	1.82	1.99	1.59	1.66	1.32	8.84
α-pinene		0.94	1.75	3.05	0.4	4.77	3.64	3.82	5.86	0.72	0	0.59	0.32	0.77	2.65	1.18	10.75
β-pinene		0.83	1.79	3.09	0.43	4.82	3.69	3.87	5.9	0.83	0.11	0.7	0.34	0.82	2.69	1.23	10.8
camphene		0.83	1.79	3.09	0.43	4.82	3.69	3.87	5.9	0.83	0.11	0.7	0.34	0.82	2.69	1.23	10.8
sabinene		1.03	1.74	3.08	0.42	4.78	3.67	3.85	5.87	0.7	0.15	0.47	0.37	0.74	2.6	1.14	10.77
α-phellandrene		1.19	1.48	2.75	0.25	4.46	3.34	3.52	5.56	0.46	0.32	0.51	0.22	0.5	2.38	0.9	10.44
α-terpinene		1.35	1.46	2.66	0.4	4.34	3.21	3.4	5.46	0.3	0.47	0.51	0.38	0.52	2.35	0.87	10.32
δ-terpinene		1.33	1.42	2.63	0.35	4.33	3.2	3.1	5.44	0.34	0.46	0.54	0.34	0.48	2.32	0.84	15.5
**Oxygenated monoterpenes**																	
camphor		1.88	1.42	2.93	0.99	4.49	3.5	3.67	5.52	1.02	1.14	0.73	1.06	0.83	1.99	0.87	10.45
borneol		2.28	0.91	1.72	1.22	3.33	2.23	2.42	4.46	0.9	1.5	1.3	1.28	0.9	1.63	0.71	9.3
α-terpineol		2.37	0.98	1.68	1.32	3.26	2.15	2.34	4.4	0.97	1.58	1.38	1.37	1.01	1.67	0.82	9.22
piperitone		1.82	1.18	2.62	0.8	4.22	3.19	3.37	5.28	0.74	1.02	0.63	0.88	0.55	1.86	0.57	10.19
**Sesquiterpenes**																	
β-caryophyllene		1.04	1.73	2.97	0.42	4.68	3.54	3.73	5.78	0.61	0.14	0.57	0.33	0.74	2.63	1.15	10.65
**Diterpenes**																	
carnosol		2.49	1.55	2.37	1.45	3.78	2.73	2.93	4.95	0.87	1.57	1.12	1.49	1.21	2.1	1.12	9.65
carnosic acid		2.99	1.69	2.05	1.94	3.29	2.26	2.46	4.48	1.38	2.1	1.72	1.98	1.66	2.09	1.48	9.09
rosmanol		2.95	1.42	1.78	1.88	3.07	2.05	2.24	4.24	1.4	2.09	1.74	1.93	1.56	1.78	1.32	8.93
epirosmanol		2.95	1.42	1.78	1.88	3.07	2.05	2.24	4.24	1.4	2.09	1.74	1.93	1.56	1.78	1.32	8.93
rosmadial		2.6	1.25	2.42	1.53	3.8	2.88	3.05	4.85	1.25	1.75	1.28	1.61	1.19	1.56	0.94	9.69
**Triterpenes**																	
betulinic acid		2.05	1.51	2.63	1.04	4.15	3.08	3.27	5.3	0.49	1.11	0.62	1.07	0.88	2.2	0.94	10.08
ursolic acid		2.12	1.54	2.6	1.11	4.11	3.03	3.23	5.26	0.53	1.18	0.71	1.14	0.94	2.22	0.99	10.02
rosmarinic acid		4.56	2.95	2.76	3.49	3.11	2.55	2.69	4.2	2.93	3.65	3.18	3.53	3.17	2.85	2.91	8.29
**Flavonoids**																	
apigenin		4.37	2.78	2.61	3.30	3.07	2.43	2.59	4.19	2.74	3.46	3.01	3.35	2.99	2.74	2.74	8.35
hispidulin		4.44	2.88	2.71	3.37	3.14	2.52	2.67	4.26	2.8	3.52	3.07	3.42	3.07	2.85	2.83	8.38
diosmetin		4.13	2.63	2.6	3.07	3.21	2.5	2.66	4.35	2.5	3.21	2.75	3.11	2.77	2.65	2.53	8.59
hesperidin		4.92	2.87	2.43	3.82	2.38	2.19	2.26	3.27	3.42	4.09	3.67	3.9	3.44	2.4	3.08	7.53
cirsimaritin		4.13	2.64	2.6	3.07	3.21	2.49	2.65	4.36	2.5	3.22	2.76	3.12	2.78	2.68	2.55	8.59
genkwanin		4.04	2.49	2.45	2.97	3.11	2.36	2.53	4.26	2.41	3.12	2.67	3.01	2.66	2.52	2.42	8.55

HSP: Relative energy difference (RED) very good solubility 0–1 (green color); medium solubility 1–3 (yellow color); poor solubility >3 (red color). CPME—cyclopentyl methyl ether, MeTHF—methyltetrahydrofuran.

**Table 2:** Conductor-like screening model for real solvents (COSMO–RS) probability of solubility (%) of bioactive compounds from rosemary for different solvents.

	Solvents	n-Hexane	Ethyl Acetate	Methyl Acetate	Ethyl Oleate	Ethanol	1-Butanol	Isopro-panol	Methanol	Limonene	α-Pinene	Cymene	β-myrcene	CPME	Dimethyl-Carbonate	MeTHF	Water
Compounds																	
**Monoterpenes**																	
β-myrcene		69.18	81.28	60.26	100.00	11.22	20.89	16.98	4.17	95.50	81.28	100.00	100.00	100.00	39.81	100.00	0.00
α-pinene		99.08	42.66	26.92	100.00	10.47	22.91	17.38	3.63	95.50	100.00	83.18	87.10	89.13	16.60	85.11	0.00
β-pinene		95.50	57.54	34.67	100.00	12.59	25.70	19.95	4.57	97.95	99.08	89.13	89.13	97.72	22.39	95.50	0.00
camphene		97.72	51.29	34.67	100.00	12.59	25.70	19.95	4.68	97.95	99.31	89.13	91.20	97.72	22.39	93.33	0.00
sabinene		87.10	66.07	46.77	100.00	13.49	25.70	20.42	5.13	99.98	93.97	95.50	95.50	100.00	30.90	100.00	0.00
α-phellandrene		87.10	63.10	44.67	100.00	12.30	24.55	19.50	4.57	100.00	95.50	95.50	95.50	100.00	28.84	100.00	0.00
β-phellandrene		83.56	69.18	48.98	100.00	12.88	25.12	19.95	4.79	100.00	93.33	95.50	97.72	100.00	32.36	100.00	0.00
**Oxygenated monoterpenes**																	
camphor		48.54	86.98	70.07	99.25	44.06	70.94	58.92	21.45	85.15	63.09	91.76	89.39	95.89	53.75	100.00	0.10
borneol		11.22	100.00	89.13	81.28	85.11	100.00	100.00	41.69	17.78	13.49	16.98	16.60	100.00	38.02	100.00	0.02
α-terpineol		11.22	75.86	57.54	50.12	60.26	87.10	77.62	30.90	20.42	14.45	20.89	20.42	97.72	31.62	100.00	0.02
piperitone		32.36	100.00	89.13	85.11	91.20	100.00	100.00	51.29	72.44	46.77	87.10	83.18	87.10	70.79	100.00	0.07
Sesquiterpenes																	
β-caryophyllene		99.95	53.70	33.11	100.00	7.94	18.62	14.13	2.34	100.00	100.00	89.13	89.54	100.00	18.20	100.00	0.00
**Diterpenes**																	
carnosol		2.86	100.00	100.00	60.81	100.00	100.00	100.00	58.48	10.59	4.67	13.46	11.99	100.00	100.00	100.00	0.00
carnosic acid		0.86	100.00	100.00	100.00	100.00	100.00	100.00	100.00	3.37	1.41	3.89	3.49	100.00	100.00	100.00	0.00
rosmanol		0.69	100.00	100.00	41.70	100.00	100.00	100.00	100.00	3.22	1.23	4.33	3.76	100.00	100.00	100.00	0.00
epirosmanol		0.69	100.00	100.00	41.70	100.00	100.00	100.00	100.00	3.22	1.23	4.33	3.76	100.00	100.00	100.00	0.00
rosmadial		0.58	100.00	100.00	38.89	100.00	100.00	100.00	96.82	4.06	1.22	6.35	5.58	100.00	100.00	100.00	0.00
**Triterpenes**																	
betulinic acid		6.64	100.00	100.00	48.56	68.99	93.73	90.54	22.83	17.90	9.08	19.53	17.24	100.00	45.69	100.00	0.00
ursolic acid		1.07	100.00	100.00	83.73	100.00	100.00	100.00	100.00	2.78	1.39	2.59	2.32	100.00	45.15	100.00	0.00
rosmarinic acid		0.00	100.00	100.00	34.92	100.00	100.00	100.00	100.00	0.07	0.01	0.13	0.11	100.00	100.00	100.00	0.02
**Flavonoids**																	
apigenin		0.00	100.00	100.00	25.70	100.00	100.00	100.00	100.00	0.02	0.00	0.03	0.03	100.00	100.00	100.00	0.18
hispidulin		0.02	100.00	100.00	25.13	100.00	100.00	100.00	100.00	0.26	0.05	0.47	0.41	100.00	100.00	100.00	0.00
diosmetin		0.00	100.00	100.00	23.44	100.00	100.00	100.00	100.00	0.02	0.00	0.03	0.03	100.00	100.00	100.00	0.07
hesperidin		0.00	100.00	100.00	0.72	100.00	100.00	100.00	100.00	0.00	0.00	0.00	0.00	100.00	100.00	100.00	0.00
cirsimaritin		0.03	100.00	100.00	13.12	100.00	100.00	100.00	100.00	0.38	0.09	0.71	0.63	100.00	100.00	100.00	0.00
genkwanin		0.03	100.00	100.00	13.48	100.00	100.00	100.00	100.00	0.35	0.08	0.65	0.57	100.00	100.00	100.00	0.00

COSMO–RS: Low probability of solubility 0–20% (red color); medium probability of solubility 20–60 % (yellow color); high probability of solubility 60–100% (green color). CPME—cyclopentyl methyl ether, MeTHF—methyltetrahydrofur.

**Table 3:** Denotation of samples, experimental design, and process parameters.

Sample	Treatment Time (min)	Voltage (kV)	Ethanol Content (%)	Stirring Time (min)	Extraction Type
**3 R0**	0	0	0	3	CE
**9 R0**	0	0	0	9	
**3 R25**	0	0	25	3	
**9 R25**	0	0	25	9	
**3 R50**	0	0	50	3	
**9 R50**	0	0	50	9	
**RN1**	3	20	50	/	HVED
**RN2**	9	20	0	/	
**RN3**	3	20	0	/	
**RN4**	3	25	0	/	
**RN5**	9	25	25	/	
**RN6**	9	20	25	/	
**RN7**	9	20	50	/	
**RN8**	9	25	50	/	
**RN9**	3	25	25	/	
**RN10**	9	25	0	/	
**RN11**	3	25	50	/	
**RN12**	3	20	25	/	
**RA1**	3	15	50	/	
**RA2**	9	15	0	/	
**RA3**	3	15	0	/	
**RA4**	3	20	0	/	
**RA5**	9	20	25	/	
**RA6**	9	15	25	/	
**RA7**	9	15	50	/	
**RA8**	9	20	50	/	
**RA9**	3	20	25	/	
**RA10**	9	20	0	/	
**RA11**	3	20	50	/	
**RA12**	3	15	25	/	

**Table 4:** International Commission on Illumination (CIE)—L*a*b* color parameters of CE and HVED treated rosemary extracts.

Sample	L*	a*	b*	C	h	∆C	∆E	∆H	Extraction Type
**3 R0**	86.01 ± 2.01	3.87 ± 0.52	35.77 ± 1.79	35.98 ± 1.03	1.46 ± 0.02	/	/	/	CE
**9 R0**	82.09 ± 3.64	7.09 ± 1.07	44.15 ± 0.61	44.72 ± 1.56	1.41 ± 0.43	/	/	/	
**3 R25**	95.13 ± 1.97	−0.77 ± 0.03	15.26 ± 3.72	15.28 ± 0.82	−1.52 ± 0.06	/	/	/	
**9 R25**	93.55 ± 2.27	−0.41 ± 0.04	20.56 ± 4.08	20.56 ± 1.35	−1.55 ± 0.15	/	/	/	
**3 R50**	95.32 ± 4.31	−1.16 ± 0.37	13.79 ± 1.06	13.84 ± 1.10	−1.49 ± 0.09	/	/	/	
**9 R50**	94.11 ± 2.55	−0.77 ± 0.15	15.79 ± 2.35	15.81 ± 2.74	−1.52 ± 0.07	/	/	/	
**RN1**	88.56 ± 1.96	−1.81 ± 1.06	43.33 ± 1.71	43.37 ± 2.67	−1.53 ± 0.16	29.53	30.31	1.03	HVED
**RN2**	80.89 ± 1.74	6.58 ± 1.21	49.65 ± 2.09	50.08 ± 1.65	1.44 ± 0.27	5.37	5.65	1.30	
**RN3**	83.86 ±0.72	4.45 ± 0.47	43.24 ± 2.52	43.47 ± 1.79	1.47 ± 0.20	7.49	7.79	0.21	
**RN4**	84.41 ± 1.82	4.38 ± 0.69	43.40 ± 1.79	43.62 ± 3.40	1.47 ± 0.13	7.64	7.81	0.28	
**RN5**	92.19 ± 4.18	−1.70 ± 0.82	28.03 ± 0.67	28.08 ± 1.06	−1.51 ± 0.06	7.52	7.70	0.98	
**RN6**	92.22 ± 2.33	−2.38 ± 0.04	28.18 ± 1.38	28.28 ± 1.75	−1.49 ± 0.00	7.72	7.98	1.55	
**RN7**	92.42 ± 1.79	−5.22 ± 0.57	36.22 ± 1.64	36.59 ± 0.89	−1.43 ± 0.14	20.79	20.98	2.27	
**RN8**	92.52 ± 0.64	−4.07 ± 0.03	32.48 ± 0.82	32.73 ± 2.07	−1.45 ± 0.01	16.93	17.09	1.73	
**RN9**	92.40 ± 2.87	−5.28 ± 0.50	37.19 ± 2.07	37.56 ± 1.46	−1.43 ± 0.03	22.28	22.55	2.17	
**RN10**	81.96 ± 2.91	6.35 ± 0.73	47.41 ± 4.23	47.83 ± 2.37	1.44 ± 0.12	3.12	3.35	1.21	
**RN11**	91.92 ± 5.01	−4.42 ± 0.06	36.02 ± 3.16	36.29 ± 1.68	−1.45 ± 0.07	22.45	22.72	0.86	
**RN12**	95.30 ± 2.69	−1.86 ± 0.07	18.18 ± 1.06	18.27 ± 0.69	−1.47 ± 0.06	3.00	3.12	0.86	
**RA1**	92.23 ± 3.15	−2.04 ± 0.00	28.69 ± 2.74	28.76 ± 1.41	−1.50 ± 0.04	14.92	15.24	0.26	
**RA2**	82.44 ± 1.82	5.79 ± 1.14	43.08 ± 1.95	43.47 ± 2.38	1.44 ± 0.19	−1.25	1.72	1.13	
**RA3**	86.00 ± 2.47	3.43 ± 0.97	36.88 ± 0.56	37.04 ± 1.06	1.48 ± 0.02	1.06	1.19	0.55	
**RA4**	84.95 ± 0.53	3.72 ± 0.38	38.07 ± 2.03	38.25 ± 0.76	1.47 ± 0.14	2.27	2.54	0.38	
**RA5**	90.25 ± 2.74	−0.73 ± 0.05	30.17 ± 1.73	30.18 ± 0.19	−1.55 ± 0.12	9.61	10.17	0.11	
**RA6**	90.89 ± 3.17	−0.76 ± 0.14	28.81 ± 2.49	28.82 ± 1.03	−1.54 ± 0.10	8.26	8.68	0.16	
**RA7**	92.36 ±1.56	−2.91 ± 0.09	29.96 ± 0.86	30.10 ± 2.07	−1.47 ± 0.06	14.29	14.44	1.05	
**RA8**	91.24 ± 2.40	−2.84 ± 0.16	33.36 ± 1.78	33.48 ± 1.56	−1.49 ± 0.15	17.67	17.92	0.83	
**RA9**	92.84 ± 3.77	−1.43 ± 0.62	24.87 ± 0.93	24.91 ± 0.59	−1.51 ± 0.08	9.63	9.90	0.14	
**RA10**	81.04 ± 1.82	5.72 ± 0.11	43.50 ± 2.00	43.87 ± 2.24	1.44 ± 0.20	−0.84	1.84	1.26	
**RA11**	94.56 ± 3.09	−2.81 ± 0.23	23.50 ± 1.37	23.67 ± 1.38	−1.45 ± 0.04	9.83	9.88	0.63	
**RA12**	93.93 ± 2.66	−1.57 ± 0.07	21.44 ± 1.08	21.50 ± 1.59	−1.50 ± 0.13	6.22	6.35	0.41	

L*—lightness from black to white; a* from green to red, and b* from blue to yellow; C—chroma; h—hue angle; ΔE—total color difference, ΔC—difference in chroma; ΔH— difference in hue.

**Table 5:** Ultra-Performance Liquid Chromatography–Tandem Mass Spectrometry (UPLC–MS/MS) analysis of extractive compounds from rosemary (measurements for CE and HVED treated samples) (ng/mL).

Sample	Apigenin	Carnosol	Diosmetin	Hydroxytyrosol	Luteolin	Oleanolic Acid	Quercetin	Rosmarinic Acid	p-Cymene	Camphor	Thymol	Carvacrol	Extraction Type
**3 R0**	44.460	0.940	115.897	0.394	180.406	/	/	13.030	0.009	0.602	0.002	0.013	CE
**9 R0**	32.818	0.869	111.415	0.104	152.254	/	/	0.408	0.059	0.038	0.002	0.001	
**3 R25**	29.996	1.849	80.044	3.241	154.296	/	0.035	0.767	/	0.007	0.032	0.002	
**9 R25**	27.342	2.410	82.583	2.983	147.022	/	/	0.761	0.070	0.003	0.002	0.002	
**3 R50**	66.946	69.323	140.454	15.749	107.979	307.057	0.481	4756.226	0.033	0.949	0.031	0.029	
**9 R50**	90.244	34.363	179.350	59.951	236.985	390.762	0.711	5100.455	0.729	0.251	0.020	0.300	
**RN1**	159.160	207.346	310.578	68.141	305.866	288.807	11.271	5797.821	0.001	0.066	0.001	0.035	HVED
**RN2**	80.659	2.548	255.637	0.362	291.207	/	/	23.421	/	0.003	1.576	/	
**RN3**	50.146	1.143	166.246	0.583	239.840	/	/	5.173	0.012	0.214	0.062	0.224	
**RN4**	60.764	1.317	218.043	0.469	399.846	/	/	2.544	0.001	0.069	0.001	0.000	
**RN5**	137.663	27.816	376.440	96.537	415.194	/	1.608	4228.058	0.010	0.238	/	/	
**RN6**	107.933	8.988	314.863	61.995	326.021	/	0.269	3591.086	0.025	0.005	0.094	0.004	
**RN7**	119.723	349.797	177.469	39.265	126.156	2091.128	1.510	5950.966	0.001	0.003	0.002	0.001	
**RN8**	164.683	117.627	335.963	72.962	304.784	325.866	7.824	5745.552	0.002	0.004	0.003	0.005	
**RN9**	123.606	303.095	191.720	37.747	122.569	2053.066	1.380	6002.350	/	0.006	0.205	0.000	
**RN10**	95.125	3.532	362.800	3.845	600.262	4.907	/	29.489	0.001	0.153	0.001	0.000	
**RN11**	116.828	195.651	194.323	38.968	124.223	1464.630	1.115	5700.140	0.001	0.015	0.001	0.000	
**RN12**	50.827	6.240	125.214	11.977	167.060	14.068	/	68.983	0.000	0.007	0.000	0.000	
**RA1**	112.850	286.709	206.963	37.852	167.721	920.212	3.707	5648.074	0.002	0.054	0.005	0.069	
**RA2**	71.536	2.555	179.178	0.377	246.834	/	/	32.988	0.032	1.776	7.304	0.011	
**RA3**	42.925	1.156	141.806	0.298	151.249	/	/	16.758	0.009	0.239	0.000	0.000	
**RA4**	42.412	0.521	140.039	0.268	181.444	/	/	1.106	0.002	0.003	0.000	0.687	
**RA5**	95.701	11.270	266.810	58.260	285.150	/	0.124	236.826	0.001	0.030	0.013	0.059	
**RA6**	73.333	7.219	207.054	18.538	197.301	/	/	9.584	0.001	0.007	0.000	0.000	
**RA7**	90.353	236.740	164.949	40.098	127.597	954.465	2.300	5829.363	0.001	/	0.000	0.000	
**RA8**	111.501	291.279	193.902	39.888	159.881	1001.253	4.224	5872.906	0.018	0.019	0.000	0.000	
**RA9**	56.649	10.003	173.879	11.916	203.449	/	/	30.652	0.019	0.137	0.000	0.000	
**RA10**	34.136	1.326	111.703	0.276	239.407	/	/	5.206	0.000	0.006	0.000	0.001	
**RA11**	76.580	157.254	131.357	26.408	98.402	757.572	1.479	5531.217	0.002	/	0.002	0.001	
**RA12**	38.301	7.088	129.120	5.393	148.453	/	0.022	21.810	0.000	0.015	0.001	/	

/—not detected.

**Table 6:** Headspace solid-phase microextraction/gas chromatography-mass spectrometry (HS–SPME/GC–MS) analysis of volatile compounds from rosemary (measurements for CE and HVED treated samples) (%).

Sample	Area (%)				Extraction Type
	Eucalyptol (RI = 1038)	Camphor (RI = 1150)	Borneol (RI = 1172)	Linalool (RI = 1103)	
**3 R0**	40.33	26.70	13.46	/	CE
**9 R0**	34.89	24.81	15.78	/	
**3 R25**	/	/	/	/	
**9 R25**	/	/	/	/	
**3 R50**	/	/	/	/	
**9 R50**	/	/	/	/	
**RN1**	/	/	/	/	HVED
**RN2**	31.04	22.8	14.73	3.46	
**RN3**	39.44	24.63	15.58	2.96	
**RN4**	32.56	22.19	13.42	2.79	
**RN5**	2.52	1.12	0.24	/	
**RN6**	2.28	0.99	0.40	/	
**RN7**	/	/	/	/	
**RN8**	/	/	/	/	
**RN9**	/	/	/	/	
**RN10**	25.66	20.88	15.99	/	
**RN11**	/	/	/	/	
**RN12**	/	/	/	/	
**RA1**	/	/	/	/	
**RA2**	28.36	25.01	15.91	1.56	
**RA3**	34.58	24.14	11.60	/	
**RA4**	30.27	24.64	9.51	/	
**RA5**	3.92	2.07	0.46	/	
**RA6**	3.20	1.68	0.61	/	
**RA7**	/	/	/	/	
**RA8**	/	/	/	/	
**RA9**	/	/	/	/	
**RA10**	30.65	37.83	6.22	1.19	
**RA11**	/	/	/	/	
**RA12**	/	/	/	/	

/—not detected.

**Table 7:** Residue levels and maximum residue levels (MRL) of pesticides (mg/kg) and metals (mg/kg) in rosemary samples.

Component		MRL (mg/kg)	Content (mg/kg)	HVED Extracts			
				RA8	RN7	RN9	RN11
**Pesticides**	Alachlor	0.02	<0.005	/	/	/	/
	Aldrin and Dieldrin (Aldrin and dieldrin combined expressed as dieldrin)	0.01	<0.002	/	/	/	/
	Captan (Sum of captan and THPI, expressed as captan)	0.06	<0.020	/	/	/	/
	DDT (sum of p,p′-DDT, o,p′-DDT, p-p′-DDE and p,p′-TDE (DDD) expressed as DDT)	0.05	<0.004	/	/	/	/
	Endosulfan (sum of alpha- and beta-isomers and endosulfan-sulphate expresses as endosulfan)	0.05	<0.002	/	/	/	/
	Endrin	0.01	<0.004	/	/	/	/
	Heptachlor (sum of heptachlor and heptachlor epoxide expressed as heptachlor)	0.01	<0.002	/	/	/	/
	Hexachlorobenzene	0.01	<0.002	/	/	/	/
	Hexachlorocyclohexane (HCH), alpha-isomer	0.01	<0.002	/	/	/	/
	Hexachlorocyclohexane (HCH), beta-isomer	0.01	<0.002	/	/	/	/
	Iprodione	0.02	<0.010	/	/	/	/
	Lindane (Gamma-isomer of hexachlorocyclohexane (HCH))	0.01	<0.002	/	/	/	/
	Methoxychlor	0.01	<0.010	/	/	/	/
	Tolylfluanid (Sum of tolylfluanid and dimethylaminosulfotoluidide expressed as tolylfluanid)	0.05	<0.020	/	/	/	/
	Vinclozolin	0.02	<0.004	/	/	/	/
**Metals**	Lead (Pb)	3.00	<0.050	/	/	/	/
	Cadmium (Cd)	1.00	<0.006	/	/	/	/
	Mercury (Hg)	0.10	0.026	/	/	/	/
	Chromium (Cr)	/	0.240	55.3	66.1	71.0	60.5
	Nickel (Ni)	/	0.322	2.10	1.10	1.20	0.950
	Manganese (Mn)	/	21.00	7.10	5.10	5.45	6.20
	Iron (Fe)	/	163	23.6	17.6	17.0	19.8
	Copper (Cu)	/	6.40	3.00	3.75	3.95	6.90
	Zinc (Zn)	/	26.0	6.65	9.10	10.7	20.5

/—no data available.

## Supplementary Materials

The following are available online. Table S1: Model statistics for prediction of compositional parameters of rosemary extracts based on the NIR spectra, Figure S1: UPLC-MS/MS chromatograms of representative extracts: (a) CE (sample 3 R25), and (b) HVED (sample RN9), Figure S2: UPLC-MS/MS chromatograms of representative extracts: (a) CE (sample 3 R0), and (b) HVED (sample RA10), and (c) chemical structure of main detected compounds, Figure S3: Box plots for the (A) content of total phenols; antioxidant activity of the samples conducted by the (B) DPPH and (C) FRAP method and the (D) yield for samples treated by CE (R) and HVED (RN & RA), Figure S4: PCA biplot for different extraction types (CE: R; HVED; RN & RA).
