# Supplementary material for: The Potential of High Voltage Discharges for Green Solvent Extraction of Bioactive Compounds and Aromas from Rosemary (Rosmarinus officinalis L.)—Computational Simulation and Experimental Methods
Source: Molecules. 2020 Aug 14;25(16):3711. doi: 10.3390/molecules25163711 (PMC7464332; doi:10.3390/molecules25163711)
Supplement: Supplementary file 1 [file molecules-25-03711-s001.pdf]

Table S1. Model statistics for prediction of compositional parameters of rosemary extracts based on the NIR spectra.

Models	Wavelength (nm)	Observed parameter	Parameters of model efficiency			
			R^2	RMSE	RPD	RER
MODEL 1	904-1699	TPC	0.999	0.227	3.888	14.576
		FRAP	0.993	50.404	2.168	10.288
		DPPH	0.997	0.545	3.399	12.972
MODEL 2	1349-1699	TPC	0.994	2.869	2.359	8.515
		FRAP	0.987	4543.942	0.024	0.114
		DPPH	0.995	0.732	2.531	9.658
MODEL 3	904-932,1349-1699	TPC	0.996	2.307	2.935	10.589
		FRAP	0.993	50.446	2.166	10.2797
		DPPH	0.997	0.54	3.431	13.093
MODEL 4	904-932	TPC	0.432	5.74	1.182	4.255
		FRAP	0.150	113.073	0.966	4.586
		DPPH	0.685	1.167	1.588	6.063

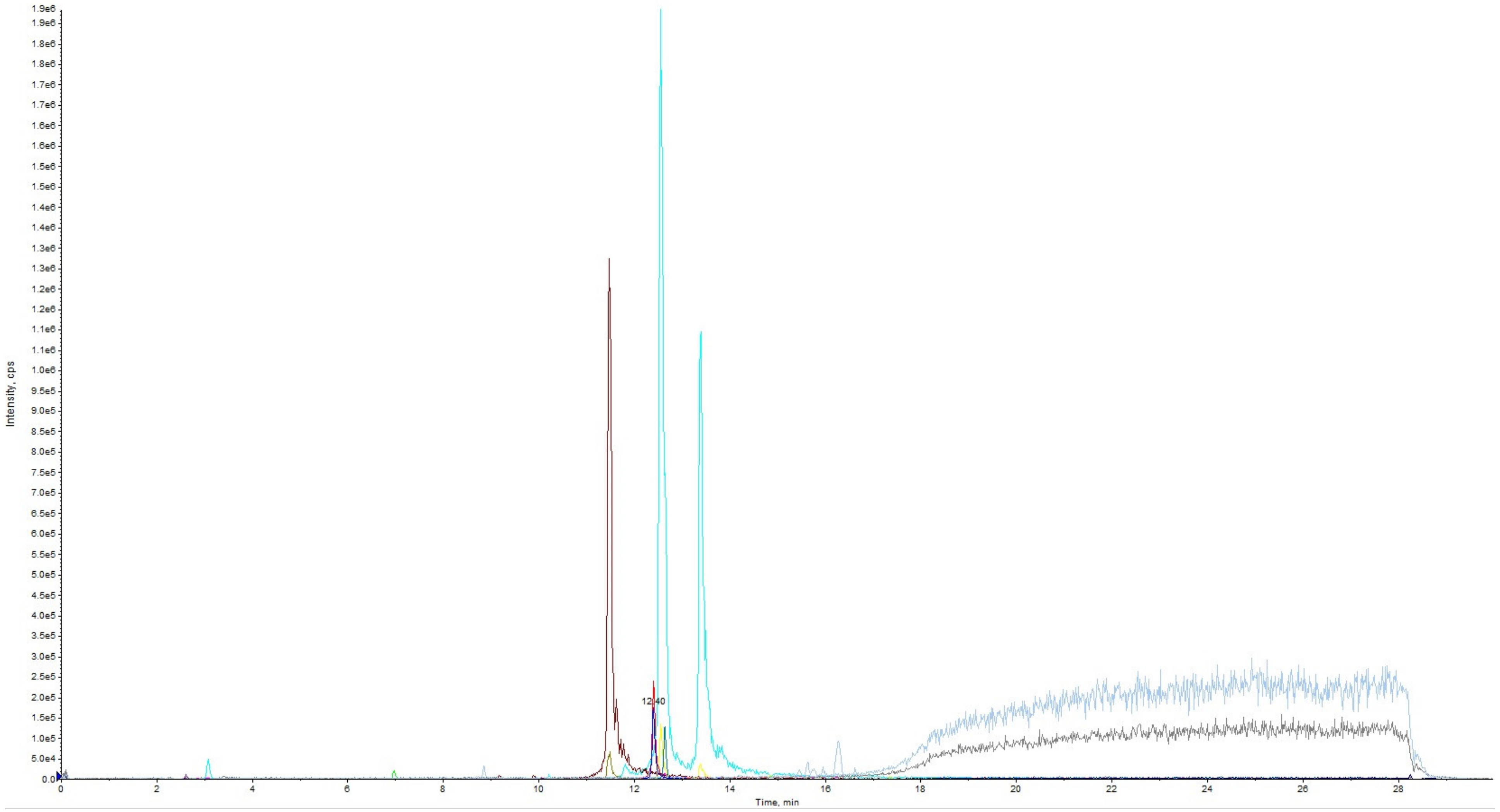

a)

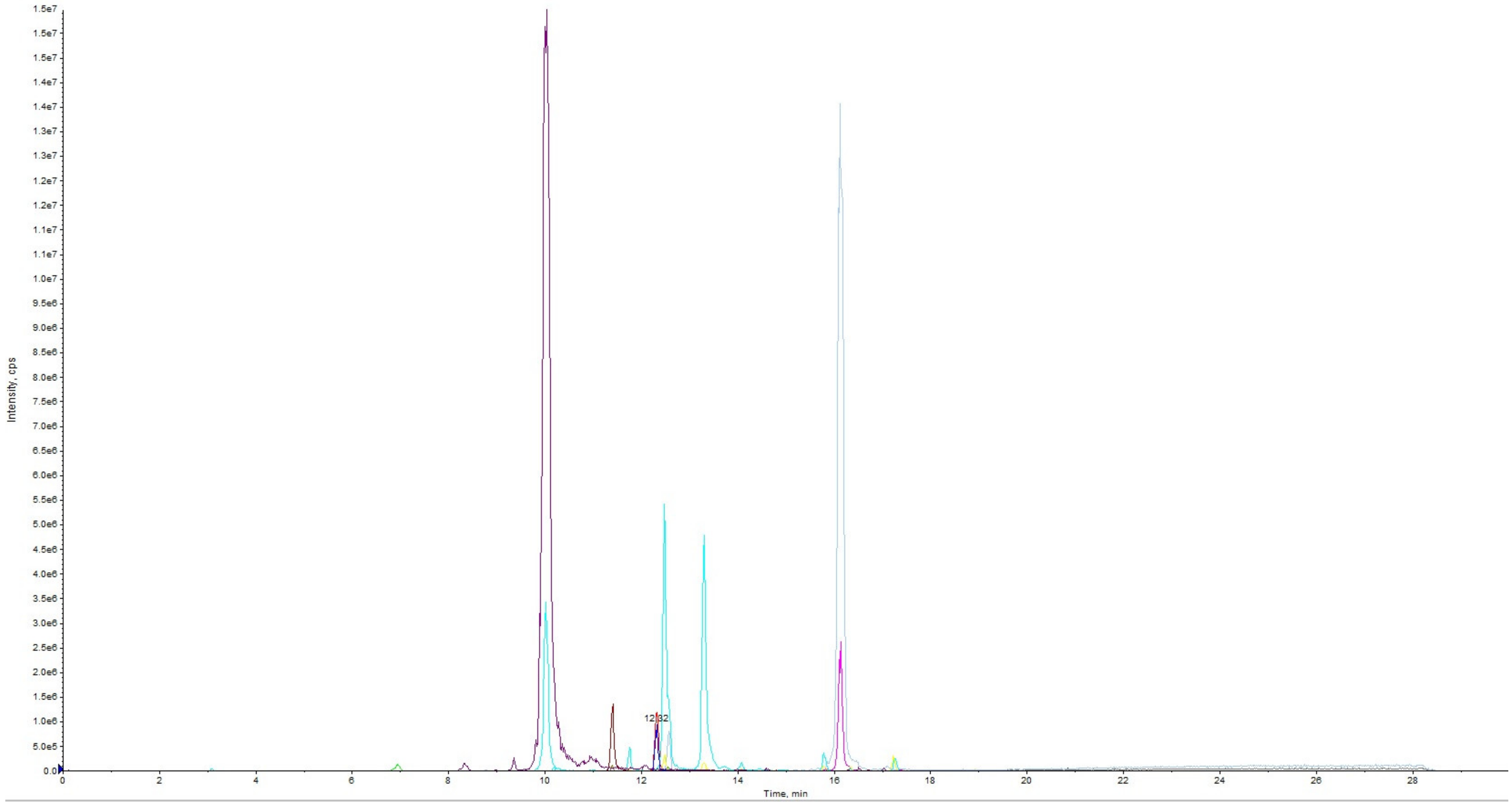

b)

Figure S1. UPLC-MS/MS chromatograms of representative extracts: a) CE (sample 3 R25), and b) HVED (sample RN9).

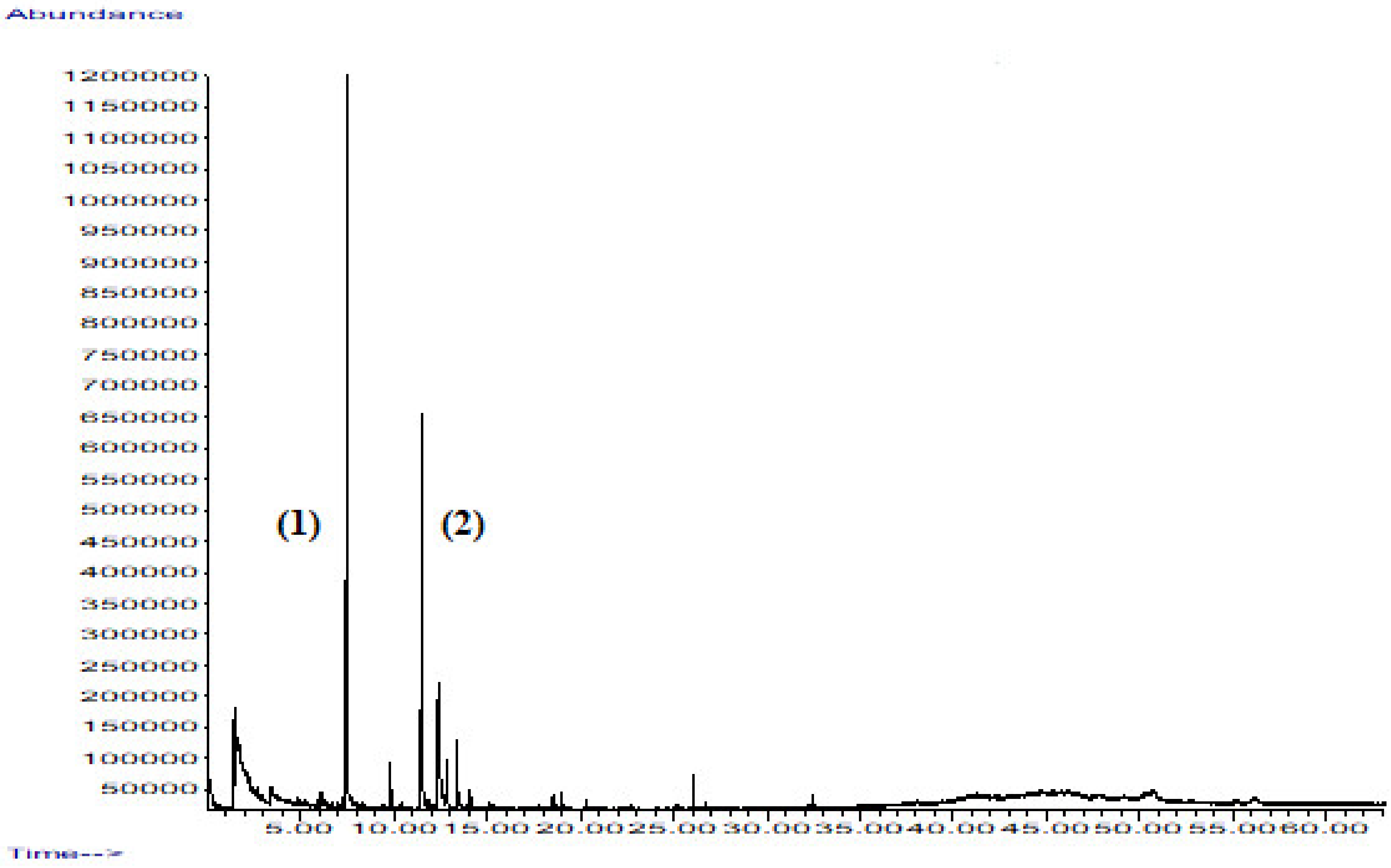
Abundance
1200000
1150000
1100000
1050000
1000000
950000
900000
850000
800000
750000
700000
650000
600000
550000
500000
450000
400000
350000
300000
250000
200000
150000
100000
50000
(1)
(2)
5.00
10.00
15.00
20.00
25.00
30.00
35.00
40.00
45.00
50.00
55.00
60.00
Time-->

a)

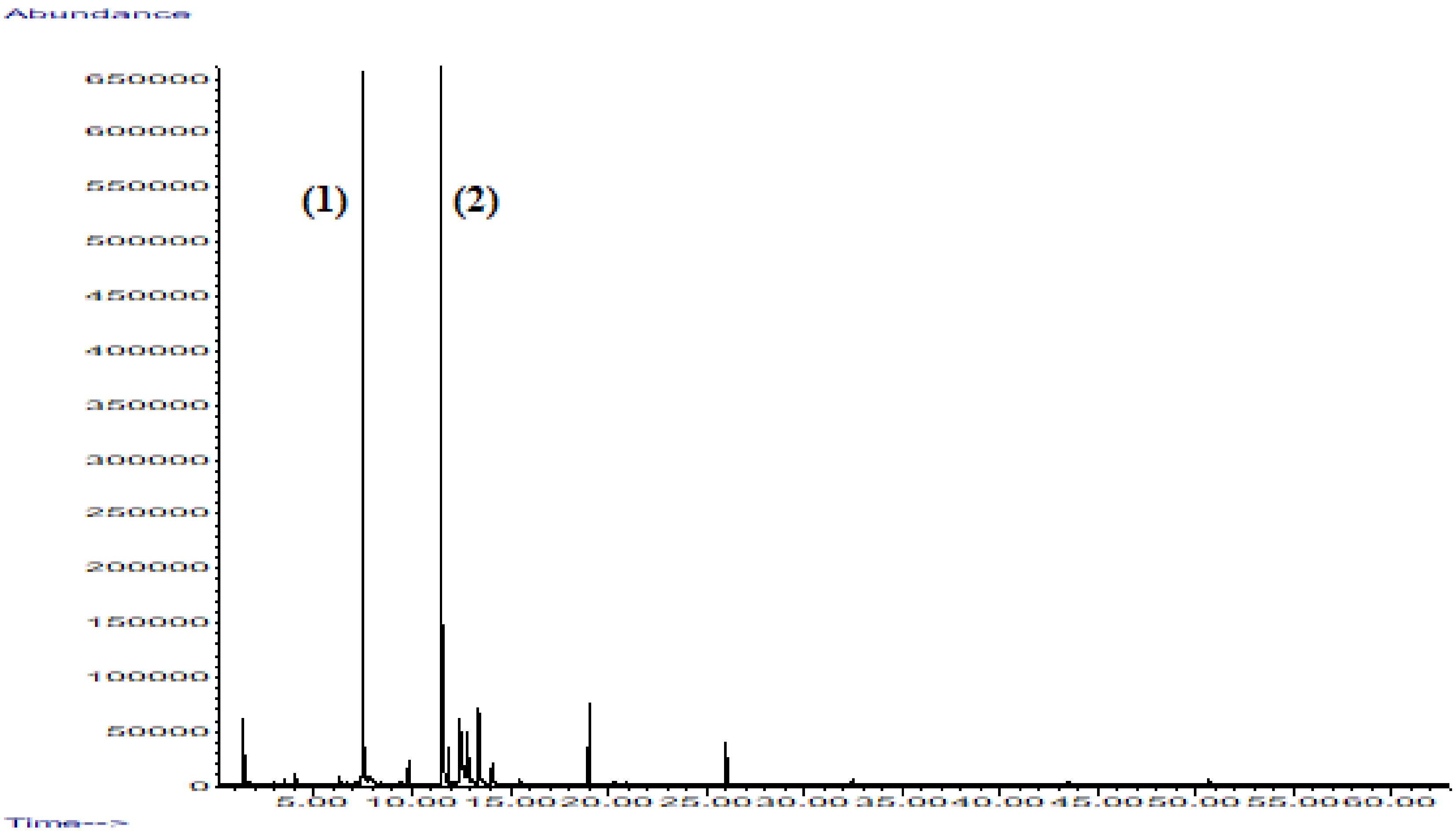
Abundance
650000
600000
550000
500000
450000
400000
350000
300000
250000
200000
150000
100000
50000
0
(1)
(2)
5.00
10.00
15.00
20.00
25.00
30.00
35.00
40.00
45.00
50.00
55.00
60.00
Time-->

b)

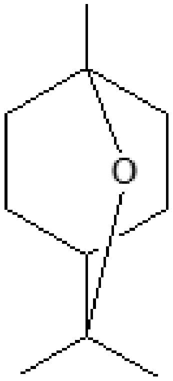

1,8-Cineole **(1)**

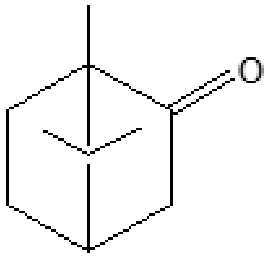

Camphor **(2)**

c)

Figure S2. UPLC-MS/MS chromatograms of representative extracts: a) CE (sample 3 R0), and b) HVED (sample RA10), and c) chemical structure of main detected compounds

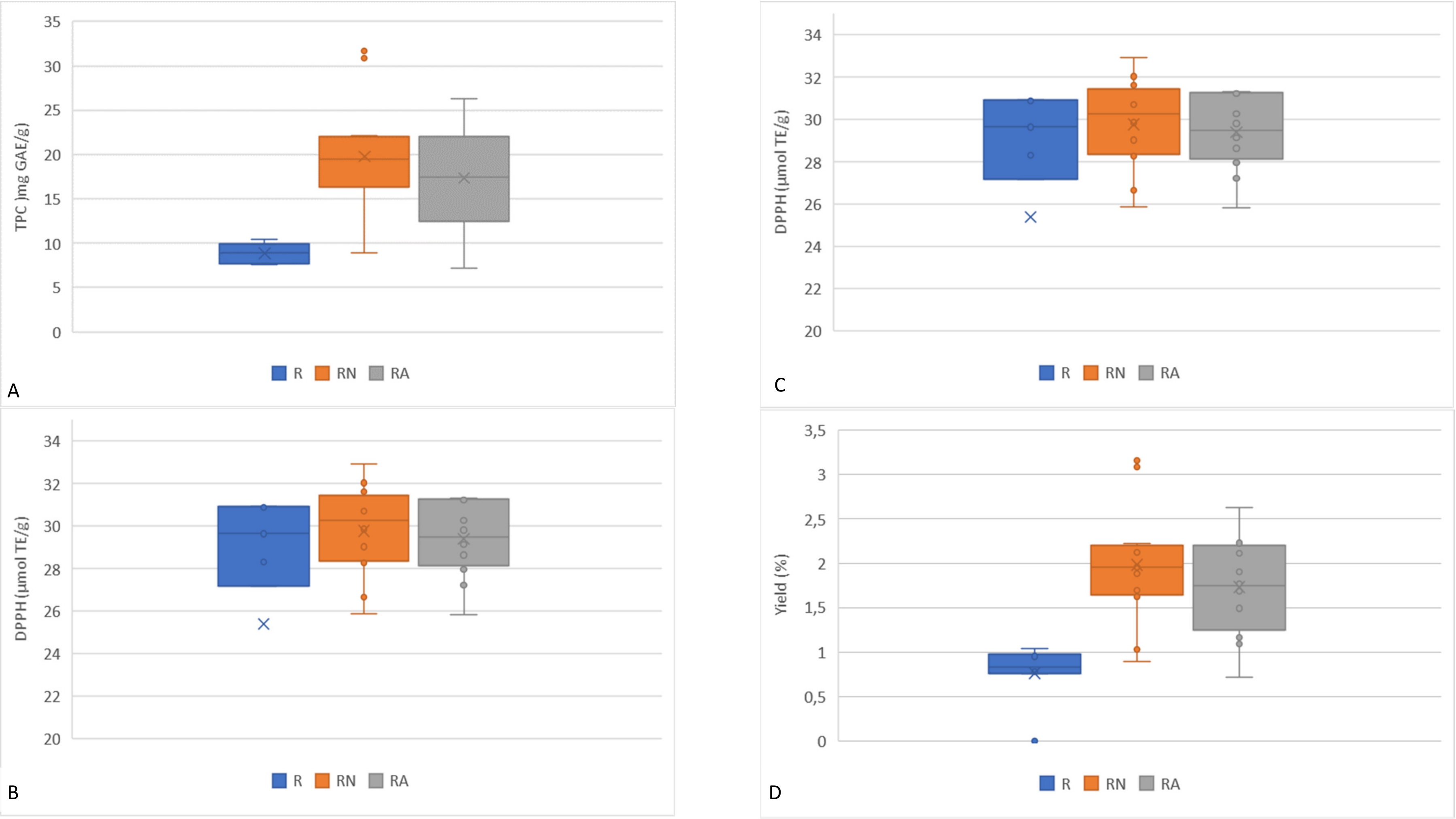

Figure S3. Box plots for the A) content of total phenols; antioxidant activity of the samples conducted by the B) DPPH and C) FRAP method and the D) yield for samples treated by CE (R) and HVED (RN & RA).

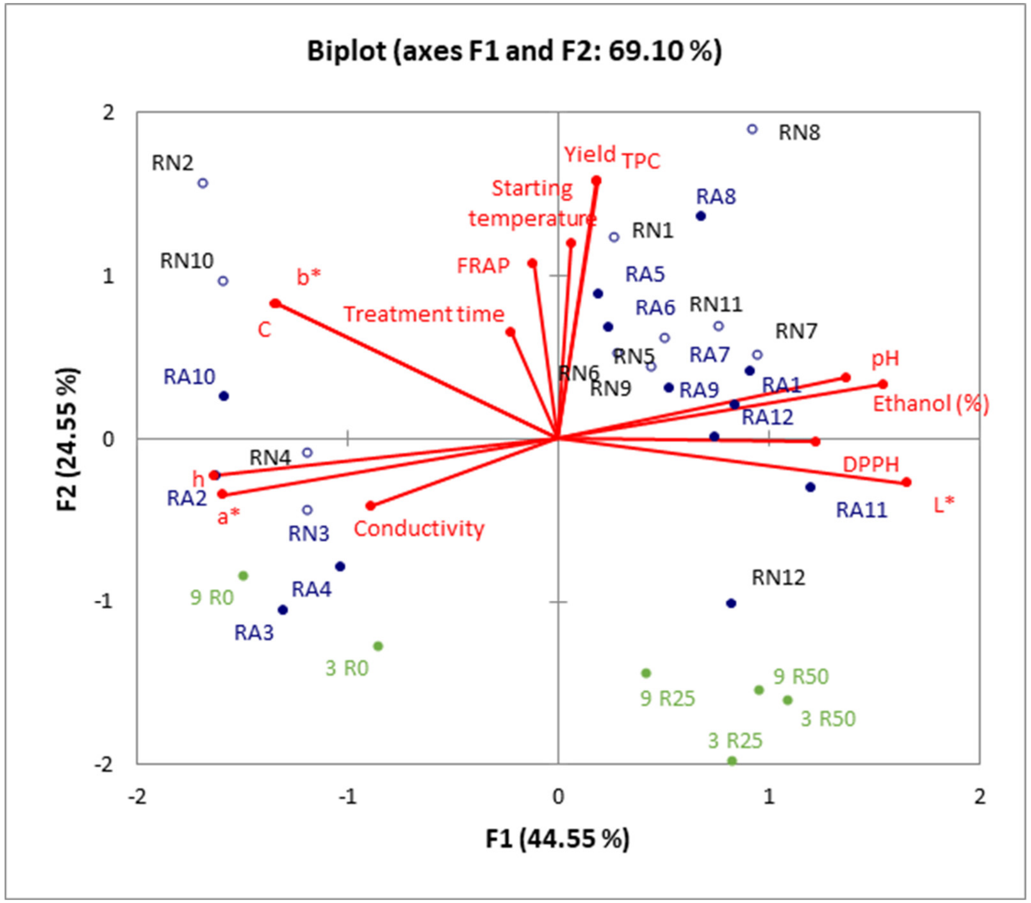

Figure S4. PCA biplot for different extraction types (CE: R; HVED; RN & RA)
